# IP3R-Mediated Compensatory Mechanism for Calcium Handling in Human Induced Pluripotent Stem Cell-Derived Cardiomyocytes With Cardiac Ryanodine Receptor Deficiency

**DOI:** 10.3389/fcell.2020.00772

**Published:** 2020-08-12

**Authors:** Xiaojing Luo, Wener Li, Karolina Künzel, Sarah Henze, Lukas Cyganek, Anna Strano, Mareike S. Poetsch, Mario Schubert, Kaomei Guan

**Affiliations:** ^1^Institute of Pharmacology and Toxicology, Technische Universität Dresden, Dresden, Germany; ^2^Clinic for Cardiology and Pneumology, Universitätsmedizin Göttingen, Göttingen, Germany; ^3^DZHK (German Center for Cardiovascular Research), Partner Site Göttingen, Göttingen, Germany

**Keywords:** ryanodine receptor 2, induced pluripotent stem cell-derived cardiomyocytes, CRISPR/Cas9, calcium handling, inositol 1,4,5-trisphosphate receptor

## Abstract

In adult cardiomyocytes (CMs), the type 2 ryanodine receptor (RYR2) is an indispensable Ca^2+^ release channel that ensures the integrity of excitation-contraction coupling, which is fundamental for every heartbeat. However, the role and importance of RYR2 during human embryonic cardiac development are still poorly understood. Here, we generated two human induced pluripotent stem cell (iPSC)-based *RYR2* knockout (RYR2^–/–^) lines using the CRISPR/Cas9 gene editing technology. We found that RYR2^–/–^-iPSCs could differentiate into CMs with the efficiency similar to control-iPSCs (Ctrl-iPSCs); however, the survival of iPSC-CMs was markedly affected by the lack of functional RYR2. While Ctrl-iPSC-CMs exhibited regular Ca^2+^ handling, we observed significantly reduced frequency and intense abnormalities of Ca^2+^ transients in RYR2^–/–^-iPSC-CMs. Ctrl-iPSC-CMs displayed sensitivity to extracellular Ca^2+^ ([Ca^2+^ ]_o_) and caffeine in a concentration-dependent manner, while RYR2^–/–^-iPSC-CMs showed inconsistent reactions to [Ca^2+^ ]_o_ and were insensitive to caffeine, indicating there is no RYR2-mediated Ca^2+^ release from the sarcoplasmic reticulum (SR). Instead, compensatory mechanism for calcium handling in RYR2^–/–^-iPSC-CMs is partially mediated by the inositol 1,4,5-trisphosphate receptor (IP3R). Similar to Ctrl-iPSC-CMs, SR Ca^2+^ refilling in RYR2^–/–^-iPSC-CMs is mediated by SERCA. Additionally, RYR2^–/–^-iPSC-CMs showed a decreased beating rate and a reduced peak amplitude of L-type Ca^2+^ current. These findings demonstrate that RYR2 is not required for CM lineage commitment but is important for CM survival and contractile function. IP3R-mediated Ca^2+^ release is one of the major compensatory mechanisms for Ca^2+^ cycling in human CMs with the RYR2 deficiency.

## Introduction

The type 2 ryanodine receptor (RYR2) represents the major sarcoplasmic reticulum (SR) Ca^2+^ release channel in adult cardiomyocytes (CMs). It plays an essential role in excitation-contraction coupling, a process by which an electrical signal is converted into a single contraction (Eisner et al., 2017). Upon spontaneous depolarization of membrane potential, voltage-dependent L-type calcium channels (LTCC) are activated, which cause the influx of a small amount of external Ca^2+^ into the cytosol. The Ca^2+^ signal is then sensed and amplified by the Ca^2+^ -sensitive RYR2. The opening of RYR2 causes a substantial release of Ca^2+^ from the SR into the cytosol and thus elevates intracellular Ca^2+^ levels. In the end, the binding of Ca^2+^ to troponin promotes sliding of thick and thin filaments, which results in cardiac contraction (Eisner et al., 2017).

A growing number of studies highlight the importance of RYR2 for the precise functionality of the adult heart. An inducible cardiac-specific *Ryr2* knockout (*Ryr2*^–/–^) mouse model claimed that RYR2 plays a non-redundant role in the control of heart rate and rhythmicity (Bround et al., 2012). An in-frame deletion of exon-3 in the *Ryr2* gene in the mouse (Ex3-del^+/−^) was associated with bradycardia and death. However, Ex3-del^+/−^ mice did not display some clinical phenotypes of patients with the RYR2 exon-3 deletion, including catecholaminergic polymorphic ventricular tachycardia (Liu et al., 2014).

In the early stage of embryonic heart development in mice, Ca^2+^ homeostasis is regulated not only by RYR2 but also by LTCC and inositol 1,4,5-trisphosphate receptor (IP3R) (Takeshima et al., 1998; Rosemblit et al., 1999; Kapur and Banach, 2007; Sasse et al., 2007). Ryr2^–/–^ mice possess the ability of repetitive Ca^2+^ signals and rhythmic contractions at embryonic day E9.5 (Takeshima et al., 1998), when LTCC and IP3R may be responsible for the early Ca^2+^ cycling (Liu et al., 2002; Kockskamper et al., 2008; Xie et al., 2018). However, as the development of the embryo proceeds, the importance of Ca^2+^ cycling via SR increases progressively (Liu et al., 2002). Ryr2^–/–^ mice died of cardiac arrest around embryonic day E10 with irregular arranged myocardium and trabeculae (Takeshima et al., 1998).

To date, studies on the expression and function of RYR2 during early embryonic cardiac development are limited to investigations using mouse Ryr2^–/–^ models and mouse embryonic stem cell-derived CMs (mESC-CMs) (Takeshima et al., 1998; Liu et al., 2002; Yang et al., 2002; Fu et al., 2006; Kapur and Banach, 2007; Sasse et al., 2007; Xie et al., 2018). The major challenge to study the origin and development of human embryonic cardiac myocytes is the limitation of a reliable human cell model, which can be used for long-term culture experiments. The appearance of human induced pluripotent stem cell (iPSC) technology has shown its great potential to solve this challenge (Takahashi et al., 2007; Yu et al., 2007). In the iPSC approach, adult somatic cells can be efficiently reprogrammed into pluripotent stem cells by ectopic expression of a set of transcription factors (Takahashi and Yamanaka, 2006). Based on this revolutionary finding, researchers have established the generation of human iPSCs and the efficient differentiation of these cells into iPSC-CMs (Itzhaki et al., 2011; Lian et al., 2013; Cyganek et al., 2018). Previous studies have demonstrated that both RYR2 and IP3R are involved in calcium handling in human ESC- and iPSC-derived CMs (Satin et al., 2008; Itzhaki et al., 2011). However, our knowledge about how these two signaling pathways are cooperated during human embryonic cardiac growth is still not sufficient.

In this study, we used iPSC technology in combination with the CRISPR/Cas9 gene editing technique to investigate the role of RYR2 in differentiation, development, and function of human iPSC-CMs. We hypothesized that the generated RYR2^–/–^-iPSC-CMs exhibit morphological and physiological abnormalities, providing insights into the function of RYR2 during human embryonic heart development.

## Materials and Methods

### Culture and Maintenance of iPSCs

Human iPSC lines iWTD2.1 and iBM76.1 were used in this study as controls (Ctrl-iPSCs), which were generated from dermal fibroblasts and mesenchymal stem cells from two healthy donors, respectively, using the STEMCCA lentivirus system, and characterized as previously described (Streckfuss-Bomeke et al., 2013; Cyganek et al., 2018). The study was approved by the Ethics Committee of the University Medical Center Göttingen (approval number: 21/1/11), and carried out in accordance with the approved guidelines. Human iPSCs were cultured in chemically defined E8 medium (Thermo Fischer Scientific) on Geltrex- (Thermo Fischer Scientific) coated cell culture plates at 37°C with 5% CO_2_. The E8 medium was changed daily and cells at ∼85% confluency were passaged using Versene (Thermo Fischer Scientific).

### Generation of RYR2^–/–^-iPSC Lines Using CRISPR/Cas9-Mediated Genome Editing

In order to generate homozygous RYR2 knockout (RYR2^–/–^) iPSC lines, two guide RNAs (gRNAs) were designed and inserted into the CRISPR/Cas9 plasmid (Sigma-Aldrich), respectively (Supplementary Figure 1A). While gRNA1 (ACGAACTCTTCGTAGTCGAGGG) targets exon 90 (g.741541-741563) of the *RYR2* gene, gRNA2 (CCTAGCCTGGTATATGACTATG) targets exon 99 (g.763747-763768). For transfection using the Amaxa Nucleofector II device (Lonza), 2 × 10^6^ iPSCs were collected and re-suspended in a mixture of 82 μl Nucleofector solution and 18 μl supplement 1 (Nucleofector kit 1/2, Lonza) containing 4 μg plasmid. One day after transfection, GFP^+^ cells were sorted and seeded on Geltrex-coated 96-well plates at a density of 1 × 10^3^ cells/well for expansion. The genomic DNA from the colonies was isolated and purified using the automated Maxwell 16 cell DNA purification kit (Promega) according to the manufacturer’s instruction. For genomic DNA sequencing, the DNA sequence of RYR2 was initially amplified by PCR using the appropriate primer set (Supplementary Table 1A). DNA sequencing of PCR products from CRISPR/Cas9-edited clones was performed by a commercial sequencing facility (Seqlab, Göttingen).

To characterize the pluripotency of the CRISPR/Cas9-edited RYR2^–/–^-iPSCs, reverse transcription-PCR analysis, immunofluorescence staining, and spontaneous differentiation *in vitro* were carried out using standard protocols as described earlier (Streckfuss-Bomeke et al., 2013). For detailed description, please see the Supplementary Material.

### Directed Differentiation of iPSCs Into Cardiomyocytes

Directed differentiation of Ctrl- and RYR2^–/–^-iPSCs into CMs (Ctrl-iPSC-CMs, RYR2^–/–^-iPSC-CMs, respectively) was induced by modulating WNT signaling as previously described (Lian et al., 2013; Cyganek et al., 2018). Briefly, when monolayer cultures of iPSCs on 12-well plates reached 80–90% confluency, differentiation was initiated by changing the E8 medium to cardio differentiation medium, which was composed of RPMI 1640 with Glutamax and HEPES (Thermo Fischer Scientific), 0.5 mg/ml human recombinant albumin (Sigma-Aldrich) and 0.2 mg/ml L-ascorbic acid 2-phosphate (Sigma-Aldrich). Cells were first treated with 4 μM of the GSK3β inhibitor CHIR99021 (Millipore) for 48 h and then with 5 μM of the WNT signaling inhibitor IWP2 (Millipore) for additional 48 h. Afterward, cells were cultured in cardio differentiation medium for another 4 days. From day 8, cells were cultivated in cardio culture medium containing RPMI 1640 with Glutamax and HEPES, supplemented with 2% B27 (Thermo Fischer Scientific). At day 20, beating iPSC-CMs were detached from the plate by incubating with 2 ml of 1 mg/ml collagenase B (Worthington Biochemical), dissolved in cardio culture medium, for 1 h at 37°C. Floating iPSC-CM sheet was gently transferred into a falcon tube and dissociated with 3 ml of 0.25% Trypsin/EDTA (Thermo Fischer Scientific) for 8 min at 37°C. Digestion was stopped by adding the double volume of the cardio digestion medium (80% cardio culture medium, 20% FCS, and 2 μM Thiazovivin). Cells were centrifuged at 200 g for 5 min, re-suspended in cardio digestion medium, and replated into Geltrex-coated 6-well plates at a density of 800,000 cells/well. Afterward, iPSC-CMs were cultured in cardio culture medium until 90 days.

### Time-Dependent Proliferation Analysis and Cell Viability Assay of iPSC-CMs

To investigate the proliferation of iPSC-CMs during long-term culture, both Ctrl- and RYR2^–/–^-iPSC-CMs were replated at a fixed density of 800,000 cells/well at day 20 post differentiation. Cell number was determined weekly for 10 weeks until CMs reached the age of 90 days. CMs from two randomly selected wells of one differentiation experiment were detached, dissociated, and quantified by counting the cell numbers with a hemocytometer and taking the average.

To assess the metabolic activity of iPSC-CMs, the MTT (3-(4,5-dimethylthiazol-2-yl)-2,5-diphenyl tetrasodium bromide) assay was performed according to the manufacturer’s instructions (MTT Kit CT02, Millipore). Briefly, iPSC-CMs at week 8 after replating were seeded in 48-well plates at a density of 60,000 cells per well, and cultured for another 2 weeks. Afterwards, 50 μg/ml MTT dissolved in 200 μl cardio culture medium was added onto the cells, which were then incubated at 37°C for 2 h. Reaction was stopped by the addition of 200 μl isopropanol supplemented with 0.04 M HCl. Samples were incubated and shaken (300 rpm) for 10 min at room temperature (RT). Absorbance of the formazan at 570 and 630 nm was measured using a plate reader (Biotek Synergy HTX). Cell viability was determined as absorbance (570–630 nm) and normalized to control group.

### Immunofluorescence Staining of iPSC-CMs

Ctrl- and RYR2^–/–^-iPSC-CMs grown on glass coverslips were fixed with 4% paraformaldehyde (PFA; Carl Roth), blocked with 1% bovine serum albumin (BSA; Sigma-Aldrich), and permeabilized with 0.1% Triton X-100 (Carl Roth). Immunofluorescence staining was performed overnight using the following primary antibodies: anti-α-actinin (1:500; mouse monoclonal, IgG1, Sigma-Aldrich), anti-Ki67 (1:400; rabbit polyoclonal, abcam), anti-IP3R (1:100; rabbit polyclonal, Merck Millipore, used for all three subtypes of IP3R), and anti-RYR2 (1:500; rabbit polyclonal, HPA020028; Sigma-Aldrich, used for the full length of RYR2). Afterward, cells were washed three times with PBS and incubated with the corresponding secondary antibodies (1:1000; anti-rabbit Alexa fluor 546, Invitrogen; anti-rabbit Alexa fluor 488, Invitrogen; anti-mouse Alexa fluor 546, Invitrogen; anti-mouse Alexa fluor 488, Invitrogen) for 1 h at RT. Nuclei were co-stained with 4’,6-diamidino-2-phenylindole (DAPI; 0.4 μg/ml; Sigma-Aldrich). Documentation was performed using fluorescence microscopy (Carl Zeiss).

### Western Blot

Both Ctrl- and RYR2^–/–^-iPSC-CMs (90 days old) were scraped off from the culture plates and cell pellets were snap-frozen into liquid nitrogen and stored at –80°C. For cell lysis, frozen cell pellets were resuspended in lysis buffer containing 20 mM Tris/HCl (pH 7.4), 200 mM NaCl, 1 mM Na_3_VO_4_, 20 mM NaF, 1% IGEPAL CA-630 (Sigma-Aldrich), 1 mM dithiothreitol (Roth), PhosSTOP phosphatase inhibitor (Roche), and cOmplete^TM^ protease inhibitor (Roche). After incubation for 30 min on ice, lysates were centrifuged and protein concentration was determined using the Pierce BCA protein assay kit (Thermo Fisher Scientific) according to the manufacturer’s instructions. A total amount of 40 μg protein lysate mixed with SDS loading buffer and DPBS in a volume of 20 μl was denatured for 30 min at 37°C. Afterward, samples were run on a 6–15% polyacrylamide gel with a 5% stacking gel at 250 mA for around 1–2 h and then transferred to PVDF membranes using the Wet/Tank blotting system. Unspecific binding sites on the membrane were blocked with 1% BSA in TBS with 0.1% Tween 20 (TBS-T) or with 5% non-fat dry milk in TBS-T for 1 h at RT. Membranes were incubated with primary antibodies overnight at 4°C. The following primary antibodies were used: anti-RYR2 (1:500; rabbit polyclonal; SAB4502707; Sigma-Aldrich, used for the detection of both the full-length and the truncated RYR2), anti-RYR2 (1:1000; mouse monoclonal, IgG1, MA3-916 C3-33; Thermo Fisher Scientific Pierce antibodies used for the detection of the full length of RYR2), anti-IP3R (1:750; rabbit polyclonal), anti-SERCA2A (sarco/endoplasmic reticulum Ca^2+^ -ATPase type 2A; 1:1000; mouse monoclonal, IgG2a, Thermo Fisher Scientific), anti-NCX1 (sodium-calcium exchanger type 1; 1:1000; mouse monoclonal, IgG2b, Novus), anti-Ca_v_1.2 (voltage-dependent L-type calcium channel alpha 1C subunit; 1:200; mouse monoclonal, IgG2b, abcam), anti-α-actinin (1:1000; mouse monoclonal, IgG1), anti-eukaryotic elongation factor 2 (EEF2; 1:50,000; rabbit polyclonal, IgG, abcam), anti-cardiac troponin T (cTNT; 1:1000; rabbit polyclonal, IgG, abcam), and anti-GAPDH (glyceraldehyde 3-phosphate dehydrogenase; 1:5000; rabbit polyclonal, Thermo Fisher Scientific). The membranes were then incubated with HRP-conjugated goat anti-mouse or anti-rabbit secondary antibodies (1:10,000; Thermo Fisher Scientific) for 1 h at RT. Afterwards, membranes were washed three times with TBS-T. Antigens of interest were detected by chemiluminescence (ECL; GE Healthcare) and visualized using the ChemiDoc MP system. Quantification was performed by calculating the signal intensity with Image Lab software (Bio-Rad).

To detect protein degradation, Ctrl-, A3 and A5 RYR2^–/–^-iPSC-CMs (90 days old) were stimulated with 100 nM isoprenaline (Sigma-Aldrich) for 6 h and stepwise treated with the proteasome and calpain inhibitor MG132 (10 μM for 24 h; Sigma-Aldrich) and the autophagy inhibitor bafilomycin A1 (BafA1; 100 nM for 6 h; Sigma-Aldrich) before the cell pellets were collected.

### Reverse Transcription-PCR Analysis

For gene expression analysis, Ctrl-iPSCs, CRISPR-edited A3 and A5 RYR2^–/–^-iPSCs as well as 90-day-old Ctrl- and RYR2^–/–^-iPSC-CMs were washed three times with PBS and cell pellets were collected, snap-frozen into liquid nitrogen, and stored at –80°C. Isolation and purification of total RNA were performed using the SV total RNA isolation system (Promega) in accordance to the manufacturer’s instructions. First strand cDNA synthesis was performed using MULV reverse transcriptase (Thermo Fisher Scientific) and Oligo d(T)16 primer (Thermo Fisher Scientific). The expression level of *SOX2, OCT4, NANOG, LIN28*, and *FOXD3* were assessed in Ctrl- and CRISPR-edited iPSC lines by reverse transcription-PCR using the GoTaq DNA polymerase (Promega). In addition, the expression level of *RYR2*, *IP3R1*, *IP3R2*, *CACNA1C*, *TNNT2*, and *ACTN2* were assessed in Ctrl and RYR2^–/–^-iPSC-CMs. GAPDH was used as an internal control. Primer sequences, annealing temperature, and cycles used for reverse transcription-PCR analyses are listed in Supplementary Table 1B.

### Calcium Spark Measurement of iPSC-CMs

For calcium spark recordings, spontaneously beating Ctrl- and RYR2^–/–^-iPSC-CMs (around 80-day-old) were dissociated, replated on Geltrex-coated coverslips at a density of 200,000 cells per 6-well and then allowed to recover for at least 10 days in cardio culture medium. Before measurement, cells were loaded with the fluorescent calcium indicator 5 μM fluo-4/AM (Thermo Fisher Scientific) and 0.02% [w/v] pluronic F-127 (Thermo Fisher Scientific) in Tyrode’s solution containing (in mM): NaCl 140, KCl 5.4, CaCl_2_ 1.8, MgCl_2_ 1, HEPES 10, and glucose 10 (pH adjusted to 7.3 with NaOH) for 30 min at RT. Following incubation, the indicator-containing solution was removed and cells were washed twice, and incubated for additional 10 min to allow de-esterification of the indicator. To detect Ca^2+^ sparks, both Ctrl- and RYR2^–/–^-iPSC-CMs were pre-treated with 100 nM isoprenaline for 10 min before starting the recordings. Recordings were obtained using a LSM 710 confocal microscopy system in line scan mode (512 pixels, 45 μm, 1057.7 Hz, 20,000 cycles). Cells were incubated in Tyrode’s solution at RT and field stimulated at 0.25 Hz for at least 20 s to bring the cytosolic calcium concentration to a steady state. Fluo-4 was excited at 488 nm and emitted fluorescence was captured at 490–540 nm. Quantification of Ca^2+^ sparks was performed using the SparkMaster plugin of ImageJ (NIH) and several key parameters were determined: spark frequency (events per 100 μm per second), amplitude (ΔF/F_0_), full duration at half maximum (FDHM), full width at half maximum (FWHM), and SR Ca^2+^ leak per cell (spark frequency × amplitude × FDHM × FWHM). As fixed criteria, sparks with minimal amplitude of 0.2 ΔF/F_0_, minimal width of 0.7 μm, and minimal duration of 7 ms were selected for detailed analysis.

### Calcium Transient Measurement of iPSC-CMs

Both Ctrl- and RYR2^–/–^-iPSC-CMs around day 80 were dissociated and replated on coverslips at a density of 200,000 cells/well. Cells were allowed to recover for at least 10 days post-replating. For measurement, cells were loaded with Fura-2 (Thermo Fisher Scientific) at a final concentration of 5 μM in cardio culture medium for 30 min at 37°C and washed twice with the medium. Prior to measurement, cells were incubated for 10 min to enable complete de-esterification of intracellular Fura-2. Intracellular Ca^2+^ events were recorded using a 40 × objective on an Olympus IX70 microscope fitted with an IonOptix system (Ionoptix, Milton, MA) at 35°C. Samples were excited at 340 and 380 nm with a switching frequency of 200 Hz and the emitted fluorescence was collected at 510 nm. The cytosolic Ca^2+^ level was measured as the ratio of fluorescence at 340 and 380 nm (340/380 nm).

Spontaneous whole-cell Ca^2+^ transients were recorded in normal Tyrode’s solution containing (in mM): NaCl 138, KCl 4, CaCl_2_ 1.8, MgCl_2_ 1, NaH_2_PO_4_ 0.33, HEPES 10, and glucose 10 (pH adjusted to 7.3 with NaOH). To normalize the Ca^2+^ transient frequency, Ctrl- and RYR2^–/–^-iPSC-CMs were field-stimulated using a MyoPacer (Ionoptix, Milton, MA) at a pacing frequency of 0.5 Hz (6 V, 10 ms). Monotonic transient analysis was performed using the LabChart Pro software (ADInstrument) and the following parameters were determined: peak amplitude of Ca^2+^ transients (the Fura-2 ratio at systole subtracted by the Fura-2 ratio at diastole), decay rate (tau), as well as duration and frequency of Ca^2+^ transients.

Calcium sensitivity analysis was performed as described previously (Jiang et al., 2007). Briefly, Ctrl- and RYR2^–/–^-iPSC-CMs loaded with Fura-2 were first exposed to 0 mM Ca^2+^ Tyrode’s solution until no spontaneous Ca^2+^ transient was detected and then continuously perfused with Tyrode’s solution containing Ca^2+^ of increasing concentrations (0, 0.1, 0.2, 0.3, 0.5, 1.0, and 2.0 mM). In the end, caffeine (10 mM; Sigma-Aldrich) was applied to confirm the activity of RYR2.

To examine the caffeine-induced Ca^2+^ release in iPSC-CMs, cells loaded with Fura-2 were washed with depolarization solution containing (in mM): NaCl 112, KCl 30, CaCl_2_ 1.8, MgCl_2_ 1, NaH_2_PO_4_ 0.33, HEPES 10, and glucose 10 (pH adjusted to 7.3 with NaOH). Cytosolic Ca^2+^ level was measured before and after continuous addition of caffeine with increasing concentrations (from 0.025 to 5.0 mM).

To investigate the role of IP3R-mediated Ca^2+^ release in Ca^2+^ handling of iPSC-CMs, spontaneous Ca^2+^ transients were recorded before and after the application of the IP3R antagonists 2-aminoethoxydiphenyl borate (2-APB, 20 μM; Tocris) and Xestospongin C (XeC, 1 μM; abcam). To determine the contribution of SERCA-mediated SR Ca^2+^ uptake to Ca^2+^ cycling in iPSC-CMs, spontaneous Ca^2+^ transients were recorded before and after the application of the SERCA inhibitor thapsigargin (5 μM; Millipore). The changes of Ca^2+^ transient amplitude and frequency caused by the addition of these inhibitors were determined.

For these experiments, we used an imaging chamber (RC-47FSLP, Warner instruments), which is furnished with a field stimulation and a built-in aspiration port. For fluid control, a 12-valve superfusion system (DAD-VM, ALA Scientific Instruments) combined with a multi-tube pre-heater (MPRE8, Cell MicroControls) was used, which together allows a gentle, rapid, and direct solution change around the measured cells. The overflow solution was removed via the aspiration port by suction with a peristaltic pump (minipuls 3, Gilson).

### Patch-Clamp Recording of iPSC-CMs

Ctrl- and RYR2^–/–^-iPSC-CMs around day 80 were enzymatically singularized into single cells and seeded on coverslips at a density of 30,000 cells per 3.5-cm dish. Whole-cell patch-clamp recording of single cells was conducted using an EPC10 amplifier (HEKA Elektronik), controlled by the PatchMaster software (HEKA Elektronik). Series resistance was compensated by 85%. Pipette potentials (*V*_Pip_) were corrected for liquid junction potentials. All experiments were performed at RT. Action potential (AP) measurement was performed under current-clamp mode without any current injection in RPMI 1640 basal medium using the pipette solution containing (in mM): K_D_-gluconate 100, KCl 60, MgATP 4, NaGTP 0.3, Na_2_-phosphocreatine 5, and HEPES 10 (pH adjusted to 7.2 with KOH). Voltage-clamp measurement of L-type calcium current (*I*_CaL_) was conducted in an extracellular solution containing (in mM): NMDG-Cl 140, CaCl_2_ 1.8, MgCl_2_ 1, HEPES 10, and glucose 10 (pH adjusted to 7.3 with HCl) and a pipette solution containing (in mM): CsCl 110, TEA-Cl 20, MgCl_2_ 5, EGTA 10, HEPES 10, and Na_2_ATP 2 (pH adjusted to 7.2 with CsOH). To elicit *I*_CaL_, cells were clamped for 600 ms from the holding potential of –90 mV to test potentials between 70 and –80 mV in steps of –10 mV. The basic cycle length was 3 s.

### Statistical Analysis

Results are presented as mean ± standard error of the mean (SEM). Statistical analysis was performed using GraphPad Prism 5 software with student’s *t*-test, the one-way ANOVA with the Dunnett’s multiple comparison test or the two-way ANOVA with Sidak’s correction for comparison of more groups and conditions. Results were considered statistically significant when the *P*-value was <0.05 (*P < 0.05, **P < 0.01, ***P < 0.001, ****P < 0.0001).

## Results

### Loss of *RYR2* Leads to the Increased Death of RYR2^–/–^-iPSC-CMs

**FIGURE 1:**
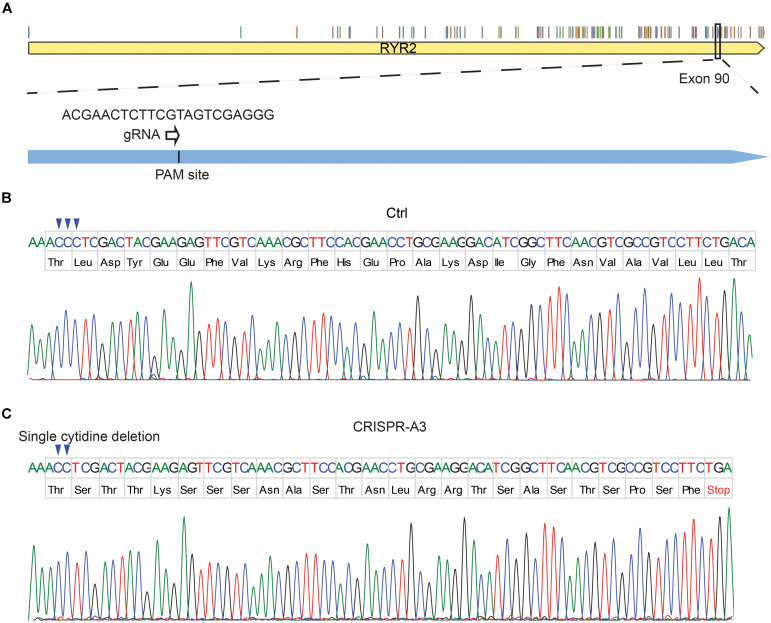
Generation of human homozygous RYR2 knockout iPSCs (RYR2^–/–^-iPSCs). **(A)** Illustration of the *RYR2* locus and gRNA1 designed for CRISPR/Cas9-mediated gene editing in exon 90 of *RYR2*. **(B,C)** DNA sequencing of Ctrl- **(B)** and CRISPR/Cas9-edited A3 RYR2^–/–^-iPSC lines **(C)**. The A3 RYR2^–/–^-iPSC line displayed a single homozygous cytidine deletion in the *RYR2* gene, which resulted in a premature termination codon (PTC).

To edit the *RYR2* gene, gRNA1 and gRNA2 were designed to target exon 90 (Figure 1A) and exon 99 (Supplementary Figure 1B) of *RYR2*, respectively. The success of the CRISPR/cas9 gene editing was screened by DNA sequencing (Figures 1B,C and Supplementary Figures 1C,D). The experiments revealed that the CRISPR/Cas9-targeted clone A3 had a homozygous deletion of one nucleotide (c.12230delC, exon 90), which resulted in a shift of the open reading frame. Consequently, the edited *RYR2* gene in A3 clone obtained a premature termination codon (PTC) in the cytosolic domain of RYR2 (L4077Sfs*25) (Figure 1C), leading to a lack of the sequence encoding transmembrane domain, which forms the channel pore. Clone A5 showed homozygous deletion of eight nucleotides (c.14159-14166del, exon 99), leading to a PTC (W4721Dfs*8; Supplementary Figure 1D), and the loss of three transmembrane helical sections and the pore-forming region.

A3 and A5 RYR2^–/–^-iPSC lines were analyzed for their pluripotency. Both cell lines were positive for the human pluripotency markers OCT4, SOX2, LIN28, NANOG, TRA-1-60, and SSEA4 as demonstrated by immunostaining (Supplementary Figure 1E), similar to their parental Ctrl-iPSCs (Cyganek et al., 2018). In addition, both A3 and A5 RYR2^–/–^-iPSC lines expressed several pluripotency genes, such as *SOX2, OCT4, NANOG, LIN28*, and *FOXD3* similar to the Ctrl-iPSCs (Supplementary Figure 1F). Differentiation potential of the edited iPSC lines *in vitro* was studied using the embryoid body-mediated differentiation method. Both A3 and A5 RYR2^–/–^-iPSCs differentiated into derivatives of three embryonic germ layers, as detected by the expression of genes encoding α-fetoprotein (*AFP*), cardiac troponin T (*TNNT2*), and synaptophysin (*SYP*) (Supplementary Figure 1G). Notably, the embryoid bodies formed from A3 and A5 RYR2^–/–^-iPSCs grew slower than those from Ctrl-iPSCs and revealed smaller sizes (data not shown).

Importantly, using the standard directed differentiation protocol, both A3 and A5 RYR2^–/–^-iPSCs were able to be successfully differentiated into spontaneously beating CMs (RYR2^–/–^-iPSC-CMs; Supplementary Videos 1, 2) similar to Ctrl-iPSCs (Supplementary Video 3). The percentage of cTNT-positive CMs at day 20 post differentiation of RYR2^–/–^-iPSCs (A3: 96.7 ± 0.5%, *n* = 3; A5: 96.6 ± 0.4%, *n* = 3) was similar to that of Ctrl-iPSCs (97.7 ± 0.8%, *n* = 3; Supplementary Figure 2A). These results indicate that loss of RYR2 does not alter the early cardiac commitment of iPSCs and the differentiation efficiency into CMs.

**FIGURE 2:**
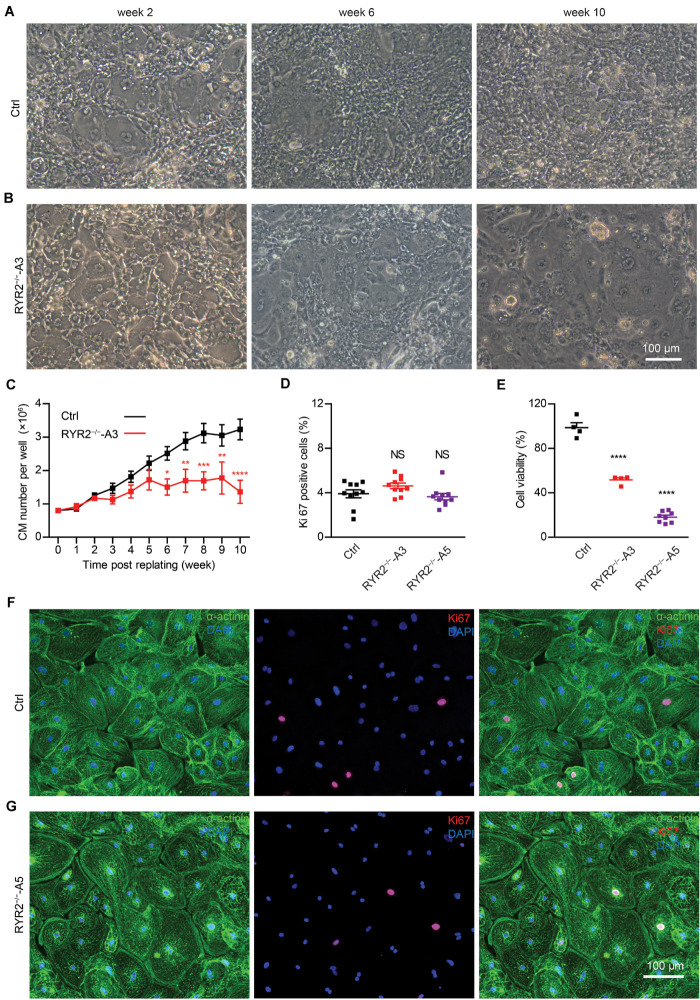
Growth analysis of Ctrl- and RYR2^–/–^-iPSC-CMs during long-term culture. **(A,B)** Bright-field images of Ctrl-iPSC-CMs **(A)** and A3 RYR2^–/–^-iPSC-CMs **(B)** after replating at day 20 of differentiation. Ctrl-iPSC-CMs showed an increase in cell density from week 2 to week 10 post replating **(A)**, while RYR2^–/–^-iPSC-CMs exhibited reduced cell density **(B)**. Scale bar, 100 μm. **(C)** Quantification of the cardiomyocyte numbers. Ctrl- and A3 RYR2^–/–^-iPSC-CMs showed distinct growth curves during long-term cell culture. Ctrl-CMs from 2 different iPSC lines and 11 differentiation experiments (iBM76.1: *n* = 6 and iWTD2.1: *n* = 5) were analyzed. A3 RYR2^–/–^-CMs from six differentiation experiments were used. *P < 0.05, **P < 0.01, ***P < 0.001, and ****P < 0.0001, RYR2^–/–^ vs. Ctrl by using the two-way ANOVA with the Sidak’s multiple comparison test. **(D)** Percentage of Ki67-positive CMs (*n* = 10 samples each for Ctrl, A3 and A5, *n* = 300–400 cells per sample counted). **(E)** Cell viability of Ctrl-, A3 and A5 RYR2^–/–^-iPSC-CMs (Ctrl: *n* = 4 from two differentiation experiments; A3 RYR2^–/–^: *n* = 4 from two differentiation experiments; A5 RYR2^–/–^: *n* = 8 from two differentiation experiments). ****P < 0.0001 by using the one-way ANOVA with the Dunnett’s multiple comparison test **(D,E)**. **(F,G)** Representative immunostaining of Ctrl- and RYR2^–/–^-iPSC-CMs probing for Ki67 (red) and α-actinin (green). Cells were counterstained with DAPI (blue) to show the nucleus. Scale bar, 100 μm.

Although RYR2^–/–^-iPSC-CMs could be cultivated for up to 3 months and remained a high percentage of cTNT-positive cells similar to the Ctrl group (Ctrl: 98.4 ± 0.2%, *n* = 3; A3: 96.8 ± 2.4%, *n* = 3; A5: 96.7 ± 0.4%, *n* = 3; Supplementary Figure 2B), they grew differently with higher number of floating cells and signs of cell death in comparison to Ctrl-iPSC-CMs (Figures 2A,B and Supplementary Figures 2C,D) during long-term culture. To analyze the survival and proliferation rate during long-term culture, both Ctrl- and RYR2^–/–^-iPSC-CMs were replated at a fixed density of 0.8 million cells/well on day 20 post differentiation. No significant difference in cell numbers was observed between Ctrl- and A3 RYR2^–/–^-iPSC-CMs during the first five weeks after replating. However, while Ctrl-iPSC-CMs revealed a steady increase in their number until week 8 and remained stable afterward, RYR2^–/–^-iPSC-CMs showed no increase in their number. At week 10, the number of Ctrl-iPSC-CMs quadrupled to 3.2 ± 0.3 million cells/well, and the number of RYR2^–/–^-iPSC-CMs only increased to 1.4 ± 0.3 million cells/well, *P* < 0.0001 (Figure 2C). To analyze whether RYR2^–/–^-iPSC-CMs have a lower proliferation capacity, Ctrl- and RYR2^–/–^-iPSC-CMs at week 8 post replating were double immunostained for sarcomeric α-actinin and Ki67, a marker of cell proliferation. The percentage of proliferating CMs was quantified as the number of Ki67-positive nuclei divided by the total number of nuclei. The number of Ki67-positive cells was comparable in Ctrl- and RYR2^–/–^-iPSC-CMs (Ctrl: 3.9 ± 0.4%; A3-RYR2^–/–^: 4.6 ± 0.3%; A5-RYR2^–/–^: 3.6 ± 0.3%; Figures 2D,F,G), suggesting that the lower cell number in RYR2^–/–^-iPSC-CMs at week 10 is not due to a change in the cell proliferation capacity. Next, we performed the MTT assay to compare the cellular metabolic activity of Ctrl- and RYR2^–/–^-iPSC-CMs, which reflects the number of viable cells presented. The results showed a significant reduction of cell viability in RYR2^–/–^-iPSC-CMs compared to Ctrl-CMs (Figure 2E).

### Loss of RYR2 Does Not Affect the Expression of Ca^2+^ Signaling-Associated Genes

**FIGURE 3:**
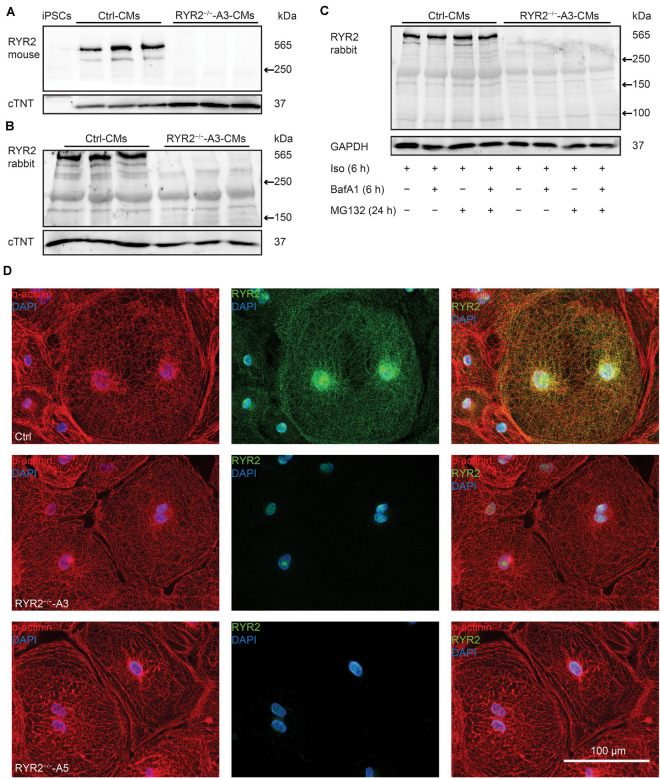
Expression of RYR2 in 3-month-old Ctrl- and RYR2^–/–^-iPSC-CMs. **(A)** Western blot analysis of the full-length RYR2 and cardiac troponin T (cTNT) in Ctrl- and A3 RYR2^–/–^-iPSC-CMs (Ctrl: *n* = 3 and A3 RYR2^–/–^: *n* = 3 independent differentiation experiments). **(B)** Western blot analysis showing protein expression of N-terminal RYR2 and cTNT in Ctrl- and A3 RYR2^–/–^-iPSC-CMs (Ctrl: *n* = 3 and A3 RYR2^–/–^: *n* = 3 independent differentiation experiments). **(C)** Representative western blot analysis of RYR2 in response to treatment with MG132 and/or Bafilomycin A (BafA1) in Ctrl- and RYR2^–/–^-iPSC-CMs (Ctrl: *n* = 3, A3 RYR2^–/–^: *n* = 3 independent differentiation experiments). No degraded RYR2 proteins were detected in RYR2^–/–^-iPSC-CMs by inhibiting protein degradation with the proteasome and calpain inhibitor MG132 and the autophagy inhibitor BafA1. **(D)** Representative immunostaining of Ctrl- and RYR2^–/–^-iPSC-CMs using antibodies against RYR2 (green) and α-actinin (red). The cells were counterstained with DAPI (blue) to show the nucleus. Scale bar, 100 μm.

**FIGURE 4:**
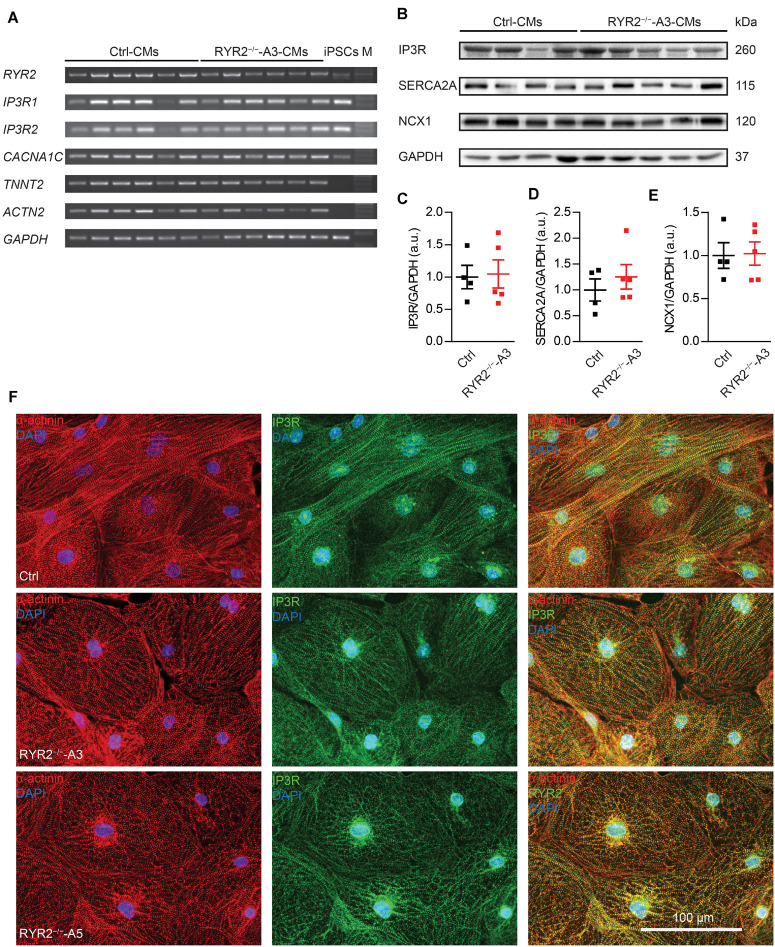
Expression of markers involved in Ca^2+^ signaling in RYR2^–/–^-iPSC-CMs compared to Ctrl-iPSC-CMs. **(A)** Reverse transcription-PCR analyses showing mRNA expression of transcripts for Ca^2+^ handling-related genes (*RYR2, IP3R1, IP3R2*, and *CACNA1C*), genes encoding sarcomeric proteins (*TNNT2* and *ACTN2*), and the housekeeping gene *GAPDH* in Ctrl- and A3 RYR2^–/–^-iPSC-CMs (Ctrl: *n* = 6 and RYR2^–/–^-A3: *n* = 6 different differentiation experiments). M: DNA molecular-weight size marker. **(B)** Western blot showing expression of Ca^2+^ handling-associated proteins (IP3R, SERCA2A, and NCX1) in both Ctrl- and A3 RYR2^–/–^-iPSC-CMs. **(C–E)** Scatter dot plot showing protein levels of IP3R **(C)**, SERCA2A **(D)**, and NCX1 **(E)** normalized to GAPDH between Ctrl- and A3 RYR2^–/–^-iPSC-CMs (Ctrl: *n* = 4 and A3 RYR2^–/–^: *n* = 5 different differentiation experiments). **(F)** Representative immunostaining of Ctrl- and RYR2^–/–^-iPSC-CMs for IP3R (green) and α-actinin (red). Cells were counterstained with DAPI (blue). Scale bar, 100 μm.

To assess whether the RYR2 expression is lost in RYR2^–/–^-iPSC-CMs, different anti-RYR2 antibodies were applied for western blot analysis. Whereas the mouse monoclonal anti-RYR2 antibody, produced by using canine cardiac Ryr2 as the immunogen, was used to detect the full-length protein of RYR2, the rabbit polyclonal anti-RYR2 antibody (SAB4502707) recognizes both the full-length and the truncated proteins by binding the N-terminal region before the PTC. Neither the full length nor the truncated proteins of RYR2 were detectable in A3 (Figures 3A,B and Supplementary Figure 3A) and A5 RYR2^–/–^-iPSC-CMs (Supplementary Figure 3B), indicating the loss of functional RYR2 in A3 and A5 RYR2^–/–^-iPSC-CMs. Immunofluorescence staining using anti-α-actinin and anti-RYR2 antibodies further confirmed the absence of RYR2 in A3 and A5 RYR2^–/–^-iPSC-CMs (Figure 3D). However, the *RYR2* mRNA levels were not altered in A3 and A5 RYR2^–/–^-iPSC-CMs compared to Ctrl-iPSC-CMs (Figure 4A and Supplementary Figure 3C). To investigate whether the absence of the truncated RYR2 in RYR2^–/–^-iPSC-CMs might be the result of protein degradation, isoprenaline-stimulated Ctrl- and RYR2^–/–^-iPSC-CMs were treated with MG132 (10 μM for 24 h) to inhibit the activity of the proteasome and with bafilomycin A1 (BafA1, 100 nM for 6 h) to inhibit the fusion of autophagosomes with lysosomes. Interference of the two major protein degradation pathways did not result in the detection of degraded RYR2 proteins in both A3 (Figure 3C) and A5 RYR2^–/–^-iPSC-CMs (Supplementary Figure 3D), suggesting that nonsense-mediated mRNA decay leads to no translation of the truncated RYR2 protein in RYR2^–/–^-iPSC-CMs.

Next, we evaluated the impact of loss of RYR2 on the expression of other cardiac Ca^2+^ signaling-associated genes in RYR2^–/–^-iPSC-CMs, such as genes encoding the cardiac IP3R types 1 and 2 (*IP3R1*, *IP3R2*) and the voltage-gated calcium channel subunit alpha-1C (*CACNA1C*). No tendency of increased or decreased expression of tested genes was observed in RYR2^–/–^-iPSC-CMs in comparison to their corresponding Ctrl-iPSC-CMs (Figure 4A and Supplementary Figure 3C). However, as RYR2^–/–^-iPSC-CMs remained the ability to spontaneously contract, we hypothesized that RYR2 may not be the only channel promoting intracellular calcium release. To verify potential compensatory mechanisms in response to loss of RYR2, we studied the expression of several Ca^2+^ -regulation related proteins. Consistent with *IP3R* mRNA levels, western blot analysis revealed no differences in IP3R protein expression in RYR2^–/–^-iPSC-CMs compared to Ctrl-iPSC-CMs. In addition, we detected no significant changes in the protein expression of SERCA2A and NCX1 between Ctrl- and RYR2^–/–^-iPSC-CMs (Figures 4B–E). To study the distribution of IP3R in iPSC-CMs, we conducted immunostaining by using antibodies against IP3R and sarcomeric α-actinin (Figure 4F). IP3R was expressed throughout the cytosol (Figure 4F middle) and partially co-localized with sarcomeric α-actinin (Figure 4F right). The perinuclear region displayed an intensive expression of IP3R. We did not observed differences in the distribution of IP3R between Ctrl- and RYR2^–/–^-iPSC-CMs.

### Loss of RYR2 Results in Significant Reduction of Spontaneous Release of Ca^2+^ Sparks in RYR2^–/–^-iPSC-CMs

**FIGURE 5:**
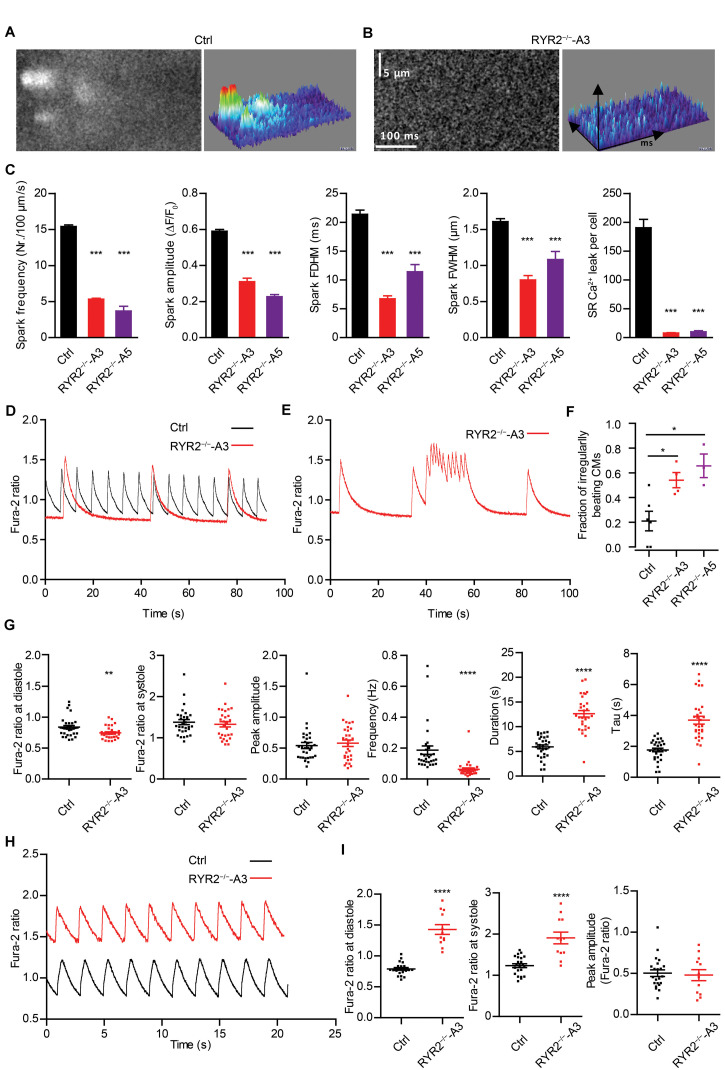
RYR2^–/–^-iPSC-CMs showed defective Ca^2+^ cycling. **(A,B)** Ca^2+^ sparks in Ctrl- and A3 RYR2^–/–^-iPSC-CMs during β-adrenergic stimulation. **(A)** Spontaneous Ca^2+^ spark images in Ctrl-iPSC-CMs (**A**; n_cell_ = 358, n_spark_ = 1521) and A3 RYR2^–/–^-iPSC-CMs (**B**; n_cell_ = 21, n_spark_ = 38) after treatment with 100 nM isoproterenol. Both Ctrl- and RYR2^–/–^-iPSC-CMs were paced at 0.25 Hz. **(C)** Bar graphs summarize the key parameters of Ca^2+^ sparks in mean ± SEM values for Ctrl- and RYR2^–/–^-iPSC-CMs: spark frequency (100 μm^–1^s^–1^), amplitude (ΔF/F_0_), FDHM (ms), FWHM (μm) and relative SR Ca^2+^ leak. For A5 RYR2^–/–^-iPSC-CMs, n_cell_ = 12, n_spark_ = 20 were analyzed. ***P < 0.001 by using the one-way ANOVA with the Dunnett’s multiple comparison test. **(D)** Representative Ca^2+^ transients in spontaneously beating Ctrl- and A3 RYR2^–/–^-iPSC-CMs. **(E)** Representative abnormal Ca^2+^ transients in A3 RYR2^–/–^-iPSC-CMs. **(F)** Fractions of irregularly beating Ctrl- and A3- and A5 RYR2^–/–^-iPSC-CMs (Ctrl: CMs from six differentiation experiments; A3 RYR2^–/–^: CMs from four differentiation experiments; A5 RYR2^–/–^: CMs from three differentiation experiments. *n* > 15 cells per experiment). *P < 0.05 by using the one-way ANOVA with the Dunnett’s multiple comparison test. **(G)** Scatter dot plot showing the diastolic Ca^2+^ levels, systolic Ca^2+^ levels, peak amplitude, frequency and duration of Ca^2+^ transients, as well as the time constant during the decay of Ca^2+^ transients (tau) in Ctrl- and A3 RYR2^–/–^-iPSC-CMs (Ctrl: *n* = 31 cells from five differentiation experiments; A3 RYR2^–/–^: *n* = 32 cells from three differentiation experiments). **(H)** Representative Ca^2+^ transients in Ctrl- and A3 RYR2^–/–^-iPSC-CMs under a normalized field stimulation at a pacing frequency of 0.5 Hz. **(I)** Scatter dot plot showing the diastolic Ca^2+^ levels, systolic Ca^2+^ levels and peak amplitude of paced Ca^2+^ transients in Ctrl- and A3 RYR2^–/–^-iPSC-CMs (Ctrl: *n* = 22 cells from three differentiation experiments; A3 RYR2^–/–^: *n* = 12 cells from two differentiation experiments). **P < 0.01; ****P < 0.0001 RYR2^–/–^ vs. Ctrl by using the unpaired Student’s *t*-test.

To determine functional consequences of the loss of RYR2, Ca^2+^ sparks were measured in Ctrl- and RYR2^–/–^-iPSC-CMs after pacing at 0.25 Hz, which were pre-treated with 100 nM isoprenaline to activate the β-adrenergic signaling and to promote the occurrence of Ca^2+^ sparks. In Ctrl-iPSC-CMs, typical Ca^2+^ sparks occurred randomly throughout the cell (Figure 5A). In contrast, Ca^2+^ sparks appeared rarely in RYR2^–/–^-iPSC-CMs (Figure 5B) and showed a significantly lower frequency with smaller amplitude (ΔF/F_0_) compared to Ctrl-iPSC-CMs (Figure 5C). Additionally, RYR2^–/–^-iPSC-CMs showed significantly reduced FDHM and FWHM, indicating smaller sizes of the released Ca^2+^ sparks in RYR2^–/–^-iPSC-CMs compared to Ctrl-iPSC-CMs. This led to a 95.7 and 94.7% reduction of spontaneous SR Ca^2+^ leak in A3 and A5 RYR2^–/–^-iPSC-CMs, respectively, compared to Ctrl-iPSC-CMs (Figure 5C). These data indicate that RYR2 plays a major role in spontaneous Ca^2+^ leak from the SR at diastole. The remaining but smaller Ca^2+^ sparks detected in RYR2^–/–^-iPSC-CMs suggest the existence of some other Ca^2+^ regulatory channels, which may mediate Ca^2+^ sparks independent of RYR2.

### RYR2^–/–^-iPSC-CMs Show Abnormal Ca^2+^ Transients Compared to Ctrl-iPSC-CMs

To further investigate whether normal Ca^2+^ homeostasis was affected in RYR2^–/–^-iPSC-CMs, spontaneous Ca^2+^ transients were assessed. Although we detected spontaneous Ca^2+^ transients in both Ctrl- and RYR2^–/–^-iPSC-CMs (Figure 5D), 54.1 ± 6.1% of A3 and 65.7 ± 9.6% of A5 RYR2^–/–^-iPSC-CMs showed abnormalities in Ca^2+^ handling (Figures 5E,F). These irregularities were mainly detectable as highly frequent Ca^2+^ release events at elevated diastolic Ca^2+^ levels (Figure 5E), which were not observed in Ctrl-iPSC-CMs. Furthermore, RYR2^–/–^-iPSC-CMs showed significantly lower diastolic Ca^2+^ levels, but no significant differences in systolic Ca^2+^ levels and peak amplitude of Ca^2+^ transients in comparison to Ctrl-iPSC-CMs (Figure 5G and Supplementary Figure 3E). We also observed a significant decrease in the frequency of Ca^2+^ transients and an increase in the duration of Ca^2+^ transients in RYR2^–/–^-iPSC-CMs compared to Ctrl-iPSC-CMs (Figure 5G and Supplementary Figure 3E), indicating that loss of RYR2 results in reduced but prolonged contraction-relaxation cycles. Moreover, the time constant (tau) during the decay of Ca^2+^ transients was significantly increased in RYR2^–/–^-iPSC-CMs compared to Ctrl-iPSC-CMs (Figure 5G and Supplementary Figure 3E). By applying a field stimulation, we normalized the Ca^2+^ cycling of Ctrl- and RYR2^–/–^-iPSC-CMs to 0.5 Hz. Paced Ca^2+^ transients in A3 RYR2^–/–^-iPSC-CMs revealed a comparable peak amplitude (Figures 5H,I). However, the diastolic level of Ca^2+^ transients after pacing was much higher in RYR2^–/–^-iPSC-CMs than in non-paced RYR2^–/–^-iPSC-CMs as well as in Ctrl-iPSC-CMs (Figures 5H,I). Together with the increased time constant of spontaneous Ca^2+^ transients in RYR2^–/–^-iPSC-CMs (Figure 5G and Supplementary Figure 3E), these data suggest that the efficiency of Ca^2+^ removal from the cytosol either by reuptake into the SR or by pumping Ca^2+^ out of the cell is lower in RYR2^–/–^-iPSC-CMs than in Ctrl-iPSC-CMs.

### The Sensitivity of RYR2^–/–^-iPSC-CMs to Extracellular Ca^2+^ and to Caffeine Is Changed

**FIGURE 6:**
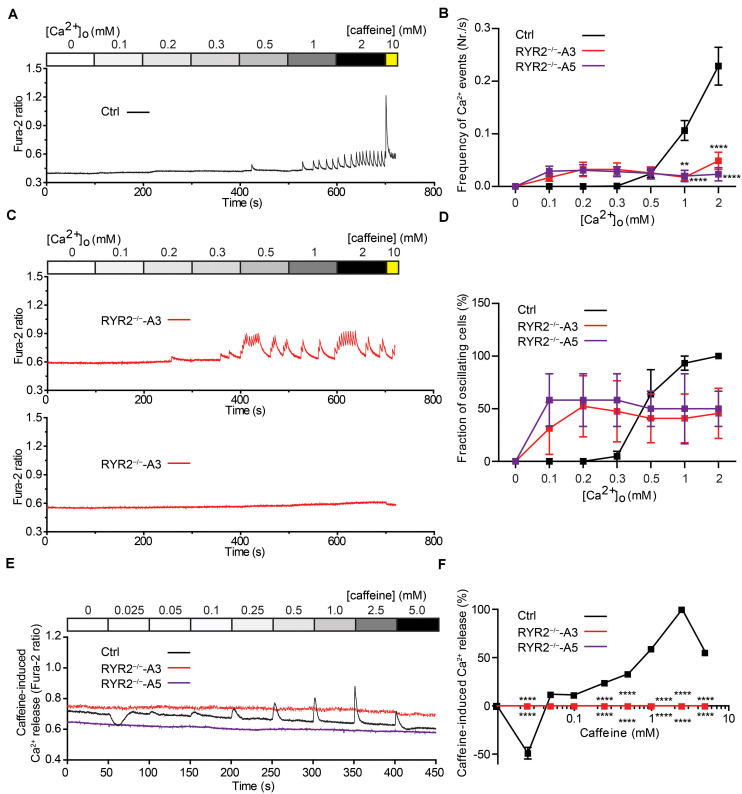
Sensitivity of Ctrl- and RYR2^–/–^-iPSC-CMs to extracellular calcium and caffeine. **(A,B)** Cytosolic Ca^2+^ dynamics in Ctrl-iPSC-CMs **(A)** and A3 RYR2^–/–^-iPSC-CMs **(B)** perfused with increasing concentrations of extracellular Ca^2+^ ([Ca^2+^ ]_o_). Cytosolic Ca^2+^ dynamics in A3 RYR2^–/–^-iPSC-CMs **(B)** were inconsistent from cell to cell. **(C)** Frequency of Ca^2+^ events detected in Ctrl- and RYR2^–/–^-iPSC-CMs under different [Ca^2+^ ]_o_. **(D)** Percentage of CMs displaying Ca^2+^ events under different [Ca^2+^ ]_o_ concentrations among the three groups (Ctrl: *n* = 20 cells from four differentiation experiments; A3 RYR2^–/–^: *n* = 15 cells from three differentiation experiments; A5 RYR2^–/–^: *n* = 12 cells from two differentiation experiments). **(E)** Representative traces of caffeine-induced Ca^2+^ release in Ctrl- and RYR2^–/–^-iPSC-CMs. **(F)** Relationship between Ca^2+^ release and caffeine concentration in CMs from the two groups. The amplitude of each caffeine-induced Ca^2+^ release was normalized to the maximum peak for each experiment (Ctrl: *n* = 20 cells from four differentiation experiments; A3 RYR2^–/–^: *n* = 6 cells from two differentiation experiments; A5 RYR2^–/–^: *n* = 6 cells from two differentiation experiments). **P < 0.01, ****P < 0.0001 RYR2^–/–^ vs. Ctrl by the two-way ANOVA with the Sidak’s multiple comparison test.

To evaluate the relationship between extracellular Ca^2+^ ([Ca^2+^ ]_o_) levels and the occurrence of Ca^2+^ transients in iPSC-CMs, we measured Ca^2+^ events under different [Ca^2+^ ]_o_ concentrations using the fluorescent Ca^2+^ dye Fura-2 in combination with Ca^2+^ imaging. To this end, spontaneously beating iPSC-CMs were first washed with Tyrode’s solution containing no Ca^2+^ until no spontaneous Ca^2+^ transient was detectable. Afterward, cytosolic Ca^2+^ changes were monitored in response to continuous perfusion with increasing Ca^2+^ concentrations (from 0 to 2.0 mM) and addition of 10 mM caffeine at the end. To quantify the fraction of Ca^2+^ -oscillating cells for each condition, the number of iPSC-CMs, which displayed spontaneous Ca^2+^ transients at each [Ca^2+^ ]_o_ concentration was determined. As shown in Figure 6A, no Ca^2+^ transients in Ctrl-iPSC-CMs were detected with [Ca^2+^ ]_o_ lower than 0.5 mM. Raising [Ca^2+^ ]_o_ concentrations stepwise from 0.3 to 2.0 mM concomitantly increased the amplitude and frequency of Ca^2+^ transients in Ctrl-iPSC-CMs (Figures 6A,C), as well as the fraction of Ca^2+^ -oscillating cells (Figure 6D). In contrast, RYR2^–/–^-iPSC-CMs responded differently to increased [Ca^2+^ ]_o_ concentrations (Figures 6B–D). While 6 out of 16 A3 RYR2^–/–^-iPSC-CMs did not display any Ca^2+^ transients from 0.1 to 2 mM [Ca^2+^ ]_o_, the remaining 10 cells revealed Ca^2+^ transients already at 0.1 or 0.2 mM [Ca^2+^ ]_o_ (Figure 6B). Similar results were observed in A5 RYR2^–/–^-iPSC-CMs; 5 out of 12 cells showed no Ca^2+^ transients under all conditions while the remaining 7 cells exhibited Ca^2+^ transients already at 0.1 mM [Ca^2+^ ]_o_ (Figure 6D). Furthermore, both A3 and A5 RYR2^–/–^-iPSC-CMs showed no caffeine-induced Ca^2+^ release as observed in Ctrl-iPSC-CMs (Figures 6A,B), which confirms the absence of functional RYR2.

Caffeine, a pharmacological agonist of RYR2 (Porta et al., 2011), is commonly used to monitor RYR2-mediated Ca^2+^ release. To determine the impact of loss of RYR2 in iPSC-CMs, we measured cytosolic Ca^2+^ levels in Ctrl- and RYR2^–/–^-iPSC-CMs before and after continuously supplementing caffeine with increasing concentrations (from 0.025 to 5.0 mM). As shown in Figure 6E, low concentration of caffeine (0.025 mM) resulted in a reduction of cytosolic Ca^2+^ levels in Ctrl-iPSC-CMs, while the amplitudes of caffeine-induced Ca^2+^ release progressively increased under the caffeine treatment at concentrations from 0.05 to 2.5 mM and declined at the concentration of 5 mM (Figure 6F). We believe that the decrease in caffeine-induced Ca^2+^ release at 5 mM caffeine might be the result of depletion of internal Ca^2+^ stores in the SR due to previous caffeine supplementation. In contrast to Ctrl-iPSC-CMs, caffeine treatment did not result in any change of cytosolic Ca^2+^ levels in both A3 and A5 RYR2^–/–^-iPSC-CMs (Figures 6E,F), indicating that no functional RYR2 is present in RYR2^–/–^-iPSC-CMs and that Ca^2+^ transients observed in RYR2^–/–^-iPSC-CMs (Figures 5D,E, 6B) are not caused by Ca^2+^ release via RYR2, rather via other mechanisms.

### IP3R-Mediated Ca^2+^ Release and SERCA-Mediated SR Ca^2+^ Uptake Are Required for the Generation of Ca^2+^ Transients in RYR2^–/–^-iPSC-CMs

**FIGURE 7:**
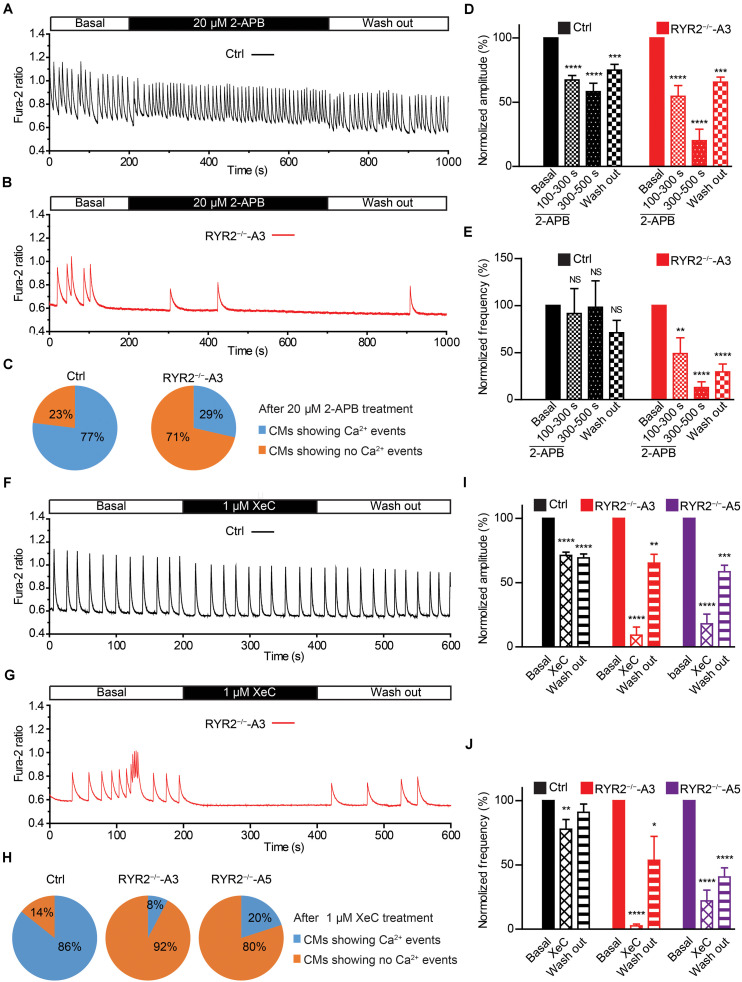
Contributions of IP3R-mediated Ca^2+^ release to spontaneous Ca^2+^ transients in Ctrl- and RYR2^–/–^-iPSC-CMs. **(A,B)** Representative cytosolic Ca^2+^ dynamics in Ctrl- **(A)** and RYR2^–/–^-iPSC-CMs **(B)** before and after the addition of IP3R antagonist 2-APB (20 μM), and after the washout of the drug. **(C)** Pie charts depict the percentage of cells maintaining Ca^2+^ transients after the treatment with 2-APB for 500 s. **(D,E)** Changes of Ca^2+^ transient amplitude **(D)** and frequency **(E)** after the treatment and the following removal of 2-APB, which were normalized to those from the same cell under the basal condition (Ctrl: *n* = 13 cells from three differentiation experiments; A3 RYR2^–/–^: *n* = 14 cells from three differentiation experiments). **(F,G)** Representative cytosolic Ca^2+^ dynamics in Ctrl- **(F)** and RYR2^–/–^-iPSC-CMs **(G)** before and after the addition of IP3R inhibitor Xestospongin C (XeC;1 μM), and after the washout of the drug. **(H)** Pie charts depict the percentage of cells maintaining Ca^2+^ transients after the treatment with XeC for 200 s. **(I,J)** Changes of Ca^2+^ transient amplitude **(I)** and frequency **(J)** after the treatment and following the washout of XeC, which were normalized to those from the same cell under the basal condition (Ctrl: *n* = 14 cells from three differentiation experiments; A3 RYR2^–/–^: *n* = 13 cells from three differentiation experiments; A5 RYR2^–/–^: *n* = 10 cells from two differentiation experiments). *P < 0.05; **P < 0.01; ***P < 0.001;****P < 0.0001 by using the one-way ANOVA with the Dunnett’s multiple comparison test.

Given that RYR2^–/–^-iPSC-CMs can spontaneously beat and release Ca^2+^ sparks and Ca^2+^ transients without RYR2 protein expression, we believe that these cells possess an alternative Ca^2+^ regulatory mechanism to compensate for the missing RYR2. IP3R-involved Ca^2+^ signaling has been discovered to play an important role during the process of cardiac development (Rosemblit et al., 1999; Poindexter et al., 2001). Therefore, we studied the effect of IP3R blockade on Ca^2+^ handling in iPSC-CMs. To this end, spontaneous Ca^2+^ transients in Ctrl- and A3 RYR2^–/–^-iPSC-CMs were measured before and after the application of 20 μM 2-APB. As shown in Figures 7A–C, 2-APB treatment for 500 s completely and reversibly blocked all Ca^2+^ transients in 10 out of 14 tested A3 RYR2^–/–^-iPSC-CMs (Figures 7B,C), while Ctrl-iPSC-CMs (10 out of 13) displayed intensive but slightly smaller Ca^2+^ transients (Figures 7A,C). Although 2-APB application decreased Ca^2+^ transient amplitude in Ctrl-iPSC-CMs (Figure 7D), it did not reduce the beating frequency of these cells (Figure 7E). On the contrary, in A3 RYR2^–/–^-iPSC-CMs, the amplitude and frequency of Ca^2+^ transients during 2-APB treatment for 100∼300 s declined to 54.7 and 49.1%, respectively, whereas the inhibitory effects increased as treatment lasted longer (300–500 s): the amplitude and frequency decreased to 20.1 and 12.9%, respectively (Figures 7D,E). Previous studies reported that 2-APB is also a blocker of store-operated Ca^2+^ entry independently of the function of IP3R (Iwasaki et al., 2001; Bootman et al., 2002). Therefore, we used the IP3R inhibitor Xestospongin C (XeC) to confirm our hypothesis. As shown in Figures 7F–J, the Ca^2+^ transients in 12 out of 13 A3 and 8 out of 10 A5 RYR2^–/–^-iPSC-CMs were completely and reversibly blocked after the addition of 1 μM XeC (Figures 7G,H), while the majority of Ctrl-iPSC-CMs (12 out of 14) still showed Ca^2+^ transients (Figures 7F,H). Additionally, RYR2^–/–^-iPSC-CMs showed a faster reaction to XeC (1 μM) than 2-APB (20 μM), as 200 s of XeC (1 μM) treatment was sufficient to block the Ca^2+^ transients in these cells. These observations imply that an IP3-gated Ca^2+^ pool is functional in iPSC-CMs, which is dominant in the modulation of Ca^2+^ handling in RYR2^–/–^-iPSC-CMs.

**FIGURE 8:**
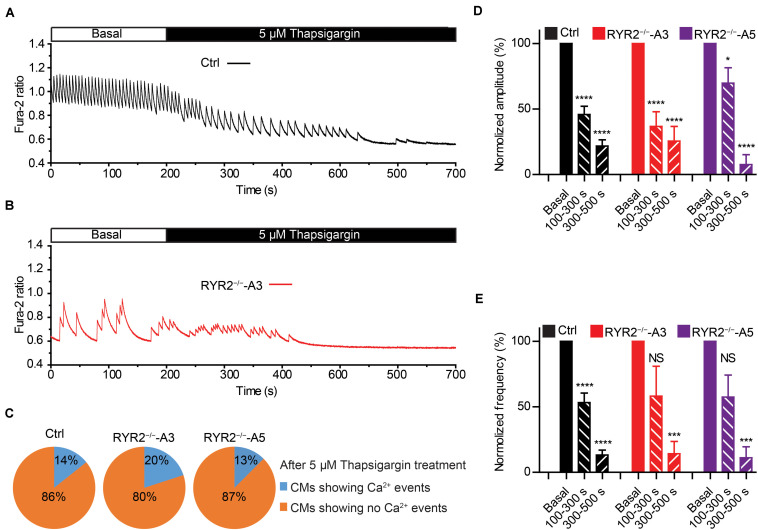
Contributions of SERCA-mediated SR Ca^2+^ uptake to spontaneous Ca^2+^ transients in Ctrl- and RYR2^–/–^-iPSC-CMs. **(A,B)** Representative cytosolic Ca^2+^ dynamics in Ctrl- **(A)** and RYR2^–/–^-iPSC-CMs **(B)** before and after the addition of SERCA inhibitor thapsigargin (5 μM). **(C)** Pie charts depict the percentage of cells maintaining Ca^2+^ transients after the treatment with thapsigargin for 500 s. **(D,E)** Changes of Ca^2+^ transient amplitude **(D)** and frequency **(E)** after the treatment with thapsigargin, which were normalized to those from the same cell under the basal condition. (Ctrl: *n* = 14 cells from three differentiation experiments; A3 RYR2^–/–^: *n* = 10 cells from three differentiation experiments; A5 RYR2^–/–^: *n* = 8 cells from two differentiation experiments). *P < 0.05; ***P < 0.001; ****P < 0.0001 by using the one-way ANOVA with the Dunnett’s multiple comparison test.

We next studied the contribution of another important Ca^2+^ handling protein located on the SR membrane, namely SERCA. To this end, we recorded spontaneous Ca^2+^ transients in Ctrl- and RYR2^–/–^-iPSC-CMs before and after the application of 5 μM thapsigargin, a specific SERCA inhibitor. It turned out that thapsigargin treatment for 500 s was sufficient to block all Ca^2+^ transients in most of Ctrl- (12 out of 14), A3 (8 out of 10) and A5 RYR2^–/–^-iPSC-CMs (7 out of 8) in a time-dependent manner (Figures 8A–E). The amplitude and frequency of Ca^2+^ transients in Ctrl-iPSC-CMs declined to 21.8 and 13.2%, respectively, during thapsigargin treatment for 300∼500 s, which was comparable with the changes found in RYR2^–/–^-iPSC-CMs (Figures 8D,E). These findings indicate that the refilling of SR Ca^2+^ content by SERCA pumps is the main mechanism in both Ctrl- and RYR2^–/–^-iPSC-CMs with no significant differences.

### RYR2^–/–^-iPSC-CMs Displayed Abnormal Action Potentials

**FIGURE 9:**
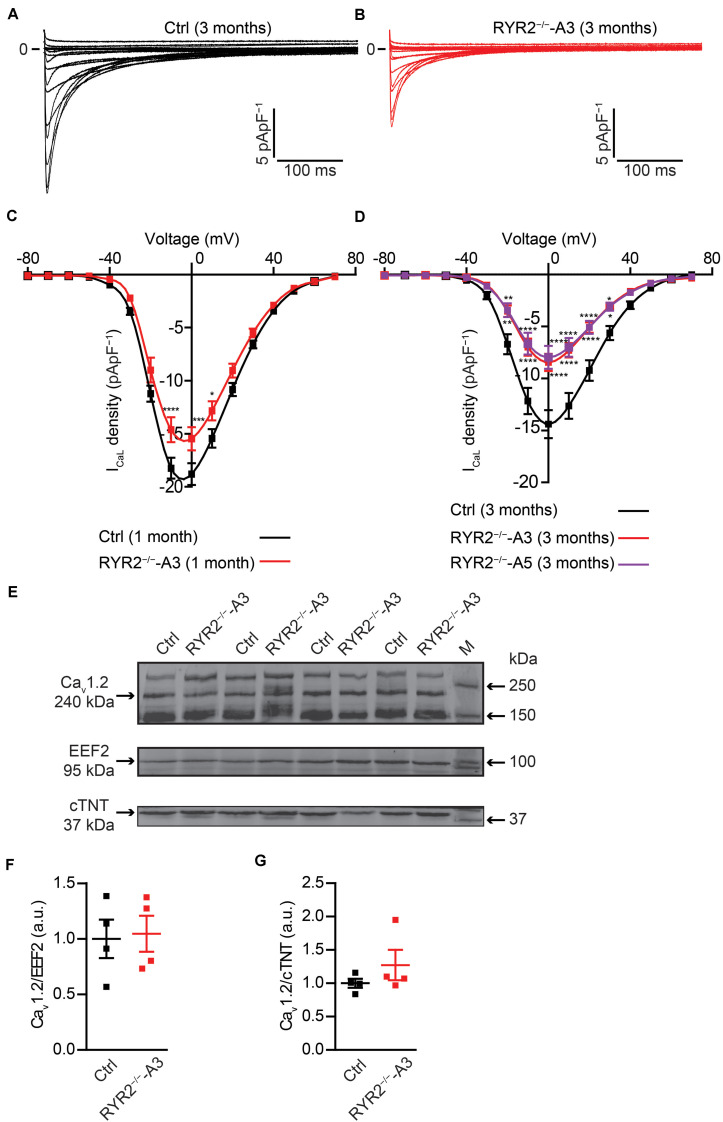
Electrophysiological analysis of *I*_CaL_ in Ctrl- and RYR2^–/–^-iPSC-CMs. **(A,B)** Representative *I*_CaL_ traces of 3-month-old Ctrl- **(A)** and A3 RYR2^–/–^-iPSC-CMs **(B)**. **(C)** Average current-voltage (I–V) curves for peak *I*_CaL_ in 1-month-old Ctrl- and RYR2^–/–^-iPSC-CMs (Ctrl: *n* = 12 cells from three differentiation experiments; RYR2^–/–^: *n* = 15 cells from two differentiation experiments). **(D)** Average current-voltage (I–V) curves for peak *I*_CaL_ in 3-month-old Ctrl- and RYR2^–/–^-iPSC-CMs (Ctrl: *n* = 32 cells from four differentiation experiments; A3 RYR2^–/–^: *n* = 24 cells from three differentiation experiments; A5 RYR2^–/–^: *n* = 18 cells from two differentiation experiments). *P < 0.05; **P < 0.01; ***P < 0.001; ****P < 0.0001 RYR2^–/–^ vs. Ctrl by the two-way ANOVA with Sidak’s multiple comparison test. **(E–G)** Western blot analysis of the voltage-dependent calcium channel alpha 1C subunit (Ca_v_1.2) in Ctrl- and A3 RYR2^–/–^-iPSC-CMs. Protein levels of Ca_v_1.2 normalized to EEF2 **(F)** and cTNT **(G)** in Ctrl- and A3 RYR2^–/–^-iPSC-CMs. Ctrl: *n* = 4 and A3 RYR2^–/–^: *n* = 4 independent differentiation experiments.

It is well-known that LTCC are the main transporter for trans-sarcolemmal Ca^2+^ influx responsible for the activation of RYR2. To understand whether loss of RYR2 function affects LTCC, we analyzed *I*_CaL_ in Ctrl- and RYR2^–/–^-iPSC-CMs. We found that *I*_CaL_ density was significantly lower in RYR2^–/–^-iPSC-CMs (Figures 9A–D). Compared to 3-month-old Ctrl-iPSC-CMs, both A3 and A5 RYR2^–/–^-iPSC-CMs at the same age presented a reduction of peak current density at 0 mV by half (Figure 9D). No significant difference in *I-V* curve of *I*_CaL_ between A3 and A5 RYR2^–/–^-iPSC-CMs was detectable. In addition, a similar tendency was also found in 1-month-old RYR2^–/–^-iPSC-CMs (Figure 9C). To investigate whether the loss of RYR2 has an effect on the protein expression of L-type Ca^2+^ channel Ca_v_1.2 in iPSC-CMs, we performed western blot analysis and found no differences between Ctrl- and RYR2^–/–^-iPSC-CMs (Figures 9E–G). These data suggest that the modulation of the LTCC (for example, calmodulin binding, or phosphorylation) might be altered in RYR2^–/–^-iPSC-CMs, leading to the inactivation of the channel or to a reduced open probability of the channel.

**FIGURE 10:**
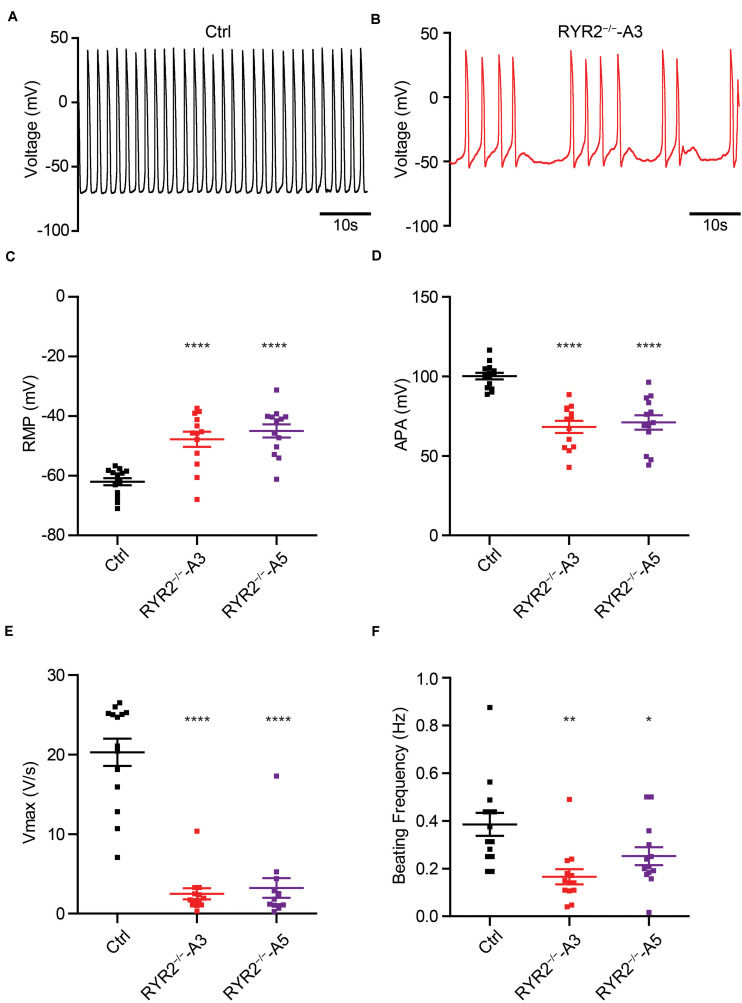
Analysis of action potentials in Ctrl- and RYR2^–/–^-iPSC-CMs. **(A,B)** Representative traces of APs revealed normal beating rhythm in Ctrl-iPSC-CMs **(A)** and arrhythmic beating in A3 RYR2^–/–^-iPSC-CMs **(B)**. **(C–F)** Scatter dot plot comparing RMP **(C)**, APA **(D)**, maximal upstroke velocity V_max_ **(E)**, and beating frequency **(F)** between Ctrl- and RYR2^–/–^-iPSC-CMs (Ctrl: *n* = 12 cells from three differentiation experiments; A3 RYR2^–/–^: *n* = 13 cells from two differentiation experiments; A5 RYR2^–/–^: *n* = 13 cells from two differentiation experiments). *P < 0.05; **P < 0.01; ***P < 0.001; ****P < 0.0001 by the one-way ANOVA with the Dunnett’s multiple comparison test.

Calcium-induced calcium release is fundamental for excitation-contraction coupling, in which RYR2 plays an important role. To investigate if RYR2^–/–^-iPSC-CMs are still able to generate cardiac action potentials (APs), we recorded APs of Ctrl- and RYR2^–/–^-iPSC-CMs at single-cell level (Figures 10A,B) and analyzed main AP parameters, including resting membrane potential (RMP), AP amplitude (APA), upstroke velocity (Vmax), and AP frequency (Figures 10C–F). While Ctrl-iPSC-CMs showed regular spontaneous APs with typical ventricular-like AP morphology, APs in both A3 and A5 RYR2^–/–^-iPSC-CMs remained mainly abnormal, as indicated by more positive RMP (Figure 10C), smaller APA (Figure 10D), reduced Vmax (Figure 10E), as well as lower and irregular beating frequency (Figures 10B,F).

## Discussion

In this study, we have generated two iPSC-based *RYR2* knockout lines using the CRISPR/Cas9 gene-editing technology to study the physiological role of RYR2 in human iPSC-CMs. Our data reveal that (i) RYR2 is not essential for the early commitment of iPSCs into the cardiac lineage, while RYR2 is important for the viability and function of iPSC-CMs; (ii) RYR2-mediated Ca^2+^ release is essential for the maintenance of normal Ca^2+^ transients, which directly modulates the beating rate of iPSC-CMs; (iii) Ca^2+^ handling in iPSC-CMs is sensitive to [Ca^2+^ ]_o_, which mainly depends on RYR2; (iv) in the absence of functional RYR2, RYR2^–/–^-iPSC-CMs generate Ca^2+^ sparks and transients via compensative IP3R-mediated pathway; and v) refilling of SR Ca^2+^ store by SERCA pumps is required for the Ca^2+^ cycling in RYR2^–/–^-iPSC-CMs similar to Ctrl-iPSC-CMs. We conclude that SR Ca^2+^ release via RYR2 is critical for the survival and whole-cell Ca^2+^ handling of iPSC-CMs. When the function of RYR2 is lost, IP3R-mediated Ca^2+^ release from the SR compensates the RYR2 function partially.

So far, the importance of RYR2 in developing fetal heart has mainly been investigated in animal models. As documented previously, the homozygous deletion of the mouse *Ryr2* gene is associated with embryonic cardiac arrest at around E10.5, even the looped heart tubes were formed at E8.5 and spontaneous heartbeat was observed at E9.5 (Takeshima et al., 1998). Cardiomyocyte-specific deletion of *Ryr2* in mice caused calpain-10 dependent programmed cell death *in vivo* (Bround et al., 2013). Moreover, homozygous deletion of exon-3 in mouse *Ryr2* gene led to embryonic lethality (Liu et al., 2014). In this study, we performed the first *in vitro* study to investigate the effect of homozygous knockout of *RYR2* on the survival and function of human iPSC-CMs that are structurally and functionally similar to human embryonic CMs (Kolanowski et al., 2017). Generated human RYR2^–/–^-iPSCs retained stem cell morphology and pluripotency and were able to differentiate into spontaneously beating CMs, which is in line with the previous study using Ryr2^–/–^mESCs (Yang et al., 2002). We detected no differences in the proliferation capacity of RYR2^–/–^-iPSC-CMs compared to Ctrl-iPSC-CMs. However, RYR2^–/–^-iPSC-CMs displayed a tremendously low cell viability during long-term culture, which resulted from increased cell death, indicating that RYR2 is critical for the survival of human embryonic CMs.

The most striking finding of the current study is that RYR2^–/–^-iPSC-CMs developed an alternative mechanism for Ca^2+^ handling to compensate for the missing of RYR2. In functional CMs, Ca^2+^ enters the cell through LTCC and activates RYR2, which results in Ca^2+^ release from the SR into the cytosol (Sham et al., 1995; Adachi-Akahane et al., 1996). It was reported that knockout of *Ryr2* in mESC-derived CMs resulted in the absence of Ca^2+^ release from the SR and an increase of *I*_CaL_ (Fu et al., 2006). Inhibition of SERCA by thapsigargin hardly affected the dynamics of Ca^2+^ transients in both *Ryr2*^–/–^ mESC-CMs and embryonic *Ryr2*^–/–^ CMs (Liu et al., 2002; Guo and Yang, 2009). Ca^2+^ transients and CM contraction in the early embryonic stage of the mouse were attained by a compensatory Ca^2+^ influx into the cytosol via LTCC, accompanied by reduced Ca^2+^ release-induced Ca^2+^ channel inactivation (Liu et al., 2002; Guo and Yang, 2009). In contrast to these data, both 1- and 3-month-old RYR2^–/–^-iPSC-CMs in the current study displayed reduced *I*_CaL_, indicating that trans-sarcolemmal Ca^2+^ influx via LTCC during each AP was lower in RYR2^–/–^-iPSC-CMs compared to Ctrl-iPSC-CMs. Moreover, we noticed that treatment of iPSC-CMs with the SERCA inhibitor thapsigargin decreased the amplitude of Ca^2+^ transients in both Ctrl- and RYR2^–/–^-iPSC-CMs in a similar time-dependent manner, resulting in a complete inhibition of Ca^2+^ transients over long time treatment. Therefore, we assume that the differences observed between human iPSC-CMs and mESC-CMs might be a result of different species. Interestingly, a recent study demonstrated that mitochondrial Ca^2+^ flux modulates the spontaneous electrical activity in *Ryr2*^–/–^ mESC-CMs, which depends on IP3R-mediated SR Ca^2+^ release (Xie et al., 2018).

IP3R shares structural and functional similarities to RYRs. IP3-operated releasable Ca^2+^ store has been demonstrated to be expressed and functional in human ESC- and iPSC-CMs (Satin et al., 2008; Sedan et al., 2008; Itzhaki et al., 2011). In our study, although IP3R gene and protein expression was not altered in RYR2^–/–^-iPSC-CMs, we discovered an increased sensitivity to the IP3R inhibitors 2-APB and XeC in iPSC-CMs. Treatment of Ctrl-iPSC-CMs with 20 μM 2-APB or 1 μM XeC, respectively, showed minor or nearly no effect on the Ca^2+^ transient frequency, while both inhibitors completely and reversibly blocked all Ca^2+^ events in more than 70% RYR2^–/–^-iPSC-CMs. These data demonstrate that the dependency of iPSC-CMs on IP3R-mediated Ca^2+^ release is significantly strengthened in the absence of functional RYR2. The finding of declined Ca^2+^ transient amplitude in Ctrl-iPSC-CMs after 2-APB application is consistent with a previous report showing that IP3-releasable Ca^2+^ pool contributes to whole-cell intracellular Ca^2+^ transients in human iPSC-CMs (Itzhaki et al., 2011). To maintain sustainable Ca^2+^ cycling, released Ca^2+^ must be removed from the cytosol. In adult human CMs, 70% of cytosolic Ca^2+^ during CM contraction is pumped back to the SR via SERCA to decrease intracellular Ca^2+^ level and to regulate SR Ca^2+^ load (Bers, 2002). SERCA-mediated Ca^2+^ uptake is required for Ca^2+^ handling in human iPSC-CMs (Itzhaki et al., 2011). These findings indicate that IP3R-mediated Ca^2+^ release and SERCA-mediated SR Ca^2+^ uptake are one of the major compensatory mechanisms for Ca^2+^ handling in human CMs when the RYR2 function is lost. Future studies should investigate whether IP3R-dependent mitochondrial Ca^2+^ flux modulates spontaneous electrical activity in human RYR2^–/–^-iPSC-CMs and whether IP3R-mediated nuclear Ca^2+^ signaling is involved in the activation of nuclear signal transduction for CM survival (Garcia and Boehning, 2017). Previous studies demonstrated that the store-operated calcium entry (SOCE) is present in embryonic cardiomyocytes and induces significant rise in Ca^2+^ entry when the depletion of SR Ca^2+^ stores occurs (Huang et al., 2006; Avila-Medina et al., 2018). In the future, it is also necessary to figure out whether SOCE plays an important role in Ca^2+^ handling in human CMs lacking functional RYR2, either by abolishing the SOCE response with known SOCE inhibitors (BTP-2 and SKF-96365) or by targeted knockdown of Orai1 with RNAi in RYR2^–/–^-iPSCs.

In the present study, we identified abnormalities of Ca^2+^ transients in more than 50% of RYR2^–/–^-iPSC-CMs. The abnormal Ca^2+^ transients were found only in a small number of Ctrl-iPSC-CMs, which is consistent with the results of other studies (Maizels et al., 2017) and may be explained by an inhomogeneous maturity of iPSC-CMs (Ronaldson-Bouchard et al., 2018). Calcium spark, an elementary event of Ca^2+^ release from SR, is believed to be mainly mediated by the spontaneous opening of one or a few RYR2 channels and contributes to the diastolic Ca^2+^ levels. In RYR2^–/–^-iPSC-CMs, β-adrenergic stimulation induced less generation of Ca^2+^ sparks with smaller size and amplitude compared to Ctrl-iPSC-CMs. This might explain the lower diastolic Ca^2+^ levels observed in spontaneously beating RYR2^–/–^-iPSC-CMs. Furthermore, Ca^2+^ transients in RYR2^–/–^-iPSC-CMs showed a comparable peak amplitude, but longer duration and slower tau compared to Ctrl-iPSC-CMs, suggesting that a higher amount of Ca^2+^ in one Ca^2+^ transient are released from the SR in RYR2^–/–^-iPSC-CMs. When RYR2^–/–^-iPSC-CMs are paced at 0.5 Hz, Ca^2+^ transients displayed comparable morphology and similar peak amplitude, but significantly elevated diastolic and systolic Ca^2+^ levels compared to Ctrl-iPSC-CMs. These findings suggest that Ca^2+^ cannot be removed rapidly and efficiently from the cytosol. Given that the SERCA-mediated refilling of SR Ca^2+^ is not significantly affected in RYR2^–/–^-iPSC-CMs, future studies should focus on the investigation of other mechanisms for Ca^2+^ removal from the cytosol, especially the function of NCX. Previous studies have shown that the majority of Ca^2+^ removal (about 75%) is accomplished by SERCA2A and by the sarcolemmal NCX (about 25%) in human cardiomyocytes. Further studies should focus on the investigation of the localization and activity of NCX in RYR2^–/–^-iPSC-CMs.

Since Ca^2+^ plays a critical role in the formation of Ca^2+^ transients, which are instrumental for subsequent cardiac contraction, we investigated the relationship between [Ca^2+^ ]_o_ levels and Ca^2+^ transients in iPSC-CMs with RYR2 deficiency. Our results revealed that 3-month-old iPSC-CMs are sensitive toward [Ca^2+^ ]_o_ at 0.5 mM or higher, with increased frequency and amplitude of Ca^2+^ transients, as well as increased fraction of Ca^2+^ -oscillating cells in response to elevated [Ca^2+^ ]_o_. These findings indicate a Ca^2+^ concentration dependent opening of RYR2 in iPSC-CMs, which is in line with the previous studies showing that human embryonic kidney (HEK) 293 cells expressing wild-type RYR2 displayed Ca^2+^ oscillations under [Ca^2+^ ]_o_ at 0.3 mM or higher and in a concentration dependent manner (Jiang et al., 2007). In contrast, RYR2^–/–^-iPSC-CMs reacted differently to the gradually increased [Ca^2+^ ]_o_ concentrations, showing Ca^2+^ transients already at very low [Ca^2+^ ]_o_ concentrations (0.1–0.2 mM), suggesting the opening of IP3R occurred at lower intracellular Ca^2+^ concentration than RYR2. In RYR2^–/–^-iPSC-CMs, spontaneous Ca^2+^ transients with lower frequency in comparison to Ctrl-iPSC-CMs were detected, which is in line with the observation in Ryr2^–/–^ mESC-CMs (Fu et al., 2006). Our data support the hypothesis by Korhonen et al. (2010) demonstrating that the developing CMs might lose the capability to generate homogeneous Ca^2+^ signals at a high frequency in response to the deficiency of the functional Ca^2+^ release channel RYR2. AP measurements revealed that loss of RYR2 resulted in a lower beating frequency in comparison to Ctrl-iPSC-CMs. These data are consistent with studies performed in mESC-derived CMs, showing similar decline in the beating frequency of Ryr2^–/–^ mESC-derived CMs (Yang et al., 2002). In addition, cardiomyocyte-specific loss of Ryr2 in mice resulted in arrhythmogenic events, such as bradycardia and tachycardia (Bround et al., 2012). As RYR2^–/–^-iPSC-CM also displayed disturbances in the rhythmicity, manifesting bradycardia, we believe that RYR2 is a critical player in the regulation of heart rate.

Furthermore, our study provides the first comprehensive assessment regarding the sensitivity of iPSC-CMs to caffeine, a pharmacological agonist of cardiac RYR2 Ca^2+^ release channels. Continuously exposing Ctrl-iPSC-CMs to caffeine with increasing concentrations resulted in a series of cytosolic Ca^2+^ changes, while low concentration of caffeine (0.025 mM) decreased cytosolic Ca^2+^ levels. Under the stimulation of 0.025 mM caffeine, HEK 293 cells expressing wild-type RYR2 presented a caffeine-induced Ca^2+^ release (Liu et al., 2010; Roston et al., 2017). This difference may be caused by the different expression and assembling of Ca^2+^ handling elements in HEK cells compared to iPSC-CMs. Interestingly, HEK 293 cells transfected with wild-type RYR2 alone or with wild-type and I4855M mutant RYR2 together showed a slight decrease of cytosolic Ca^2+^ levels at 0.025 mM caffeine (Liu et al., 2010; Roston et al., 2017), which were normally considered an artifact. However, this phenomenon was not found in RYR2^–/–^-iPSC-CMs, suggesting that the decrease of cytosolic Ca^2+^ levels might be related to RYR2 opening. We assume that such a low concentration of caffeine might result in a small amount of Ca^2+^ release from the SR in Ctrl-iPSC-CMs, which might reach a threshold of NCX activation, leading to slow extrusion of calcium from the cytosol via NCX. Further study should be performed to investigate whether the NCX inhibitor can block the reduction of cytosolic Ca^2+^ levels at a low concentration of caffeine in Ctrl-iPSC-CMs. Importantly, no changes in cytosolic Ca^2+^ levels were detected in RYR2^–/–^-iPSC-CMs treated with caffeine at low or high concentrations, which indicate that IP3R-mediated SR Ca^2+^ release in RYR2^–/–^-iPSC-CMs is not sensitive to caffeine.

## Conclusion

In conclusion, we generated two homozygous RYR2^–/–^-iPSC lines with normal capacity to differentiate into spontaneously beating CMs. However, RYR2^–/–^-iPSC-CMs in long-term culture showed enhanced cell death, indicating that RYR2 is critical for the survival but less important for the initiation of contraction of iPSC-CMs. Moreover, our study demonstrates that RYR2 plays a vital role in the maintenance of rhythmic heart beating and normal Ca^2+^ handling. Loss of functional RYR2 strengthens the dependency of iPSC-CMs on IP3R-related pathway, which combines with SERCA pumps to make critical contributions to the Ca^2+^ cycling in RYR2^–/–^-iPSC-CMs. As RYR2 is crucial for the development and function of CMs, the availability of human RYR2^–/–^-iPSC lines represent a useful tool for the *in vitro* study of early human heart development and molecular mechanism of Ca^2+^ cycling in human CMs.

## Data Availability Statement

All datasets generated for this study are included in the article/Supplementary Material.

## Ethics Statement

The studies involving the use of human induced pluripotent stem cells were reviewed and approved by the Ethics Committee of the University Medical Center Göttingen (approval number: 21/1/11), and carried out in accordance with the approved guidelines. The patients/participants provided their written informed consent to participate in this study.

## Author Contributions

XL, WL, and KG conceived the study and designed experiments, contributed to interpretation of the data, and wrote the manuscript. XL, WL, KK, SH, LC, AS, MP, and MS performed experiments and acquisition of the data. XL, WL, KK, SH, AS, MS, and KG analyzed the data. All authors contributed to the article and approved the submitted version.

## Conflict of Interest

The authors declare that the research was conducted in the absence of any commercial or financial relationships that could be construed as a potential conflict of interest.
