# Supplementary material for: IP3R-Mediated Compensatory Mechanism for Calcium Handling in Human Induced Pluripotent Stem Cell-Derived Cardiomyocytes With Cardiac Ryanodine Receptor Deficiency
Source: Front Cell Dev Biol. 2020 Aug 12;8:772. doi: 10.3389/fcell.2020.00772 (PMC7434870; doi:10.3389/fcell.2020.00772)
Supplement: Supplementary file 1 [file Data_Sheet_1.pdf]

Supplementary Data

IP3R-mediated compensatory mechanism for calcium handling in human induced pluripotent stem cell-derived cardiomyocytes with cardiac ryanodine receptor deficiency

Xiaojing Luo[a,§], Wener Li[a,§], Karolina Künzel[a], Sarah Henze[b], Lukas Cyganek[b,c], Anna Strano[a], Mareike S. Poetsch[a], Mario Schubert[a], Kaomei Guan[a,b,*]

Materials and methods

Immunofluorescent staining of iPSCs

RYR2$^{-/-}$-iPSCs grown on glass coverslips were fixed with 4% paraformaldehyde (PFA; Carl Roth) and blocked with 1% bovine serum albumin (BSA; Sigma-Aldrich). Immunofluorescence staining was performed overnight using the following primary antibodies: OCT4 (1:40; goat polyclonal, R&D systems), SOX2 (1:50; goat polyclonal, R&D systems), NANOG (1:50; goat polyclonal, abcam), LIN28 (1:300; goat polyclonal, R&D systems), stage-specific embryonic antigen 4 (SSEA4; 1:200; mouse monoclonal, abcam), and TRA-1-60 (1:200; mouse monoclonal, abcam). Afterwards, cells were washed three times with PBS and incubated with the corresponding secondary antibodies (for OCT4, SOX2, NANOG, and LIN28: 1:1000; anti-goat Alexa fluor 555, Thermo Fisher Scientific; and for SSEA4, and TRA-1-60: 1:1000; anti-mouse Alexa fluor 488, Invitrogen) for 1 hour at room temperature (RT). Nuclei were co-stained with 4',6-diamidino-2-phenylindole (DAPI; 0.4 µg/ml; Sigma-Aldrich). For antibodies against OCT4, SOX2, NANOG, and LIN 28 staining, the samples were initially permeabilized with 0.1% Triton X-100 (Carl Roth).

Spontaneous differentiation of iPSCs in vitro

To investigate the differentiation potential of the CRISPR/Cas9-edited iPSC lines, spontaneous differentiation using the embryonic body (EB) method was performed. Briefly, iPSCs were singularized using Versene and cultured in 96 U-well plate with a density of 50,000 cells per well for the EB formation. To initiate differentiation, the formed EBs were cultured for seven day in differentiation medium, composed of Iscove's modified Dulbecco's medium (Thermo Fisher Scientific), 20% FCS (Sigma-Aldrich), 1× non-essential amino acid (Thermo Fisher Scientific) and 450 µM monothioglycerol (Sigma-Aldrich). EBs at day 8 were transferred into 0.1% gelatin-coated dishes and cultivated in differentiation medium for another 10 days. Tissue-specific genes were analyzed in EB outgrowths at different differentiation stages by RT-PCR.

Flow cytometry

For flow cytometry analysis of iPSC-CMs, cells singularized with 0.25% Trypsin/EDTA were fixed with 4% PFA at RT for 10 minutes, permeabilized with 0.1% Triton X-100, and blocked with 1% BSA at 4°C for at least 2 hours. Cells were then incubated with the antibody against cTNT

(1:500; mouse monoclonal, Thermo Fisher Scientific) at 4°C overnight. After 2 times washing with PBS, samples were incubated with the corresponding secondary antibody (1:1000; anti-mouse Alexa fluor 488, Invitrogen) at RT for 1 hour, followed by another 2 times washing with PBS. Subsequently, cells resuspended in PBS were analyzed using the LSRII flow cytometry (BD Biosciences). Unstained cells and cells stained with secondary antibody only were used as negative controls. For every sample, at least 10,000 events were analyzed.

Supplementary Table 1. Primers used in this study.

a) Primers for DNA sequencing of CRISPR/Cas9-edited iPSCs.

$RYR2^{-/-}$-iPSC lines	gRNA binding region	Sequencing primers
A3	Exon 90	For: TCTTCCAACAACGTGGAGAT<br>Rev: TAATTCAGGACGCTCTCTGC
A5	Exon 99	For: AACTTTGTATCTTTTAACAAATCTCTCCCT<br>Rev: TATTTTCAGTTAATTTATCCAAAGTCAAGC

b) Primer sequences, annealing temperature, and cycles used for reverse transcription-PCR analyses.

Gene	Primer	Length (bps)	TA (°C)	Cycles
SOX2	For: ATGCACCGCTACGACGTGA<br>Rev: CTTTTGCACCCCTCCCATTT	437	60	30
OCT4	For: AGTTTGTGCCAGGGTTTTTG<br>Rev: ACTTCACCTTCCCTCCAACC	113	59	36
FOXD3	For: GTGAAGCCGCCTTACTCGTAC<br>Rev: CCGAAGCTCTGCATCATGAG	353	61	38
LIN28	For: AGTAAGCTGCACATGGAAGG<br>Rev: ATTGTGGCTCAATTCTGTGC	410	52	36
NANOG	For: AGTCCCAAAGGCAAACAACCCACTTC<br>Rev: ATCTGCTGGAGGCTGAGGTATTTCTGTCTC	164	64	36
GAPDH	For: AGAGGCAGGGATGATGTTCT<br>Rev: TCTGCTGATGCCCCCATGTT	258	60	30
AFP	For: ACTCCAGTAAACCCTGGTGTTG<br>Rev: GAAATCTGCAATGACAGCCTCA	255	60	33
TNNT2	For: GACAGAGCGGAAAAGTGGGA<br>Rev: TGAAGGAGGCCAGGCTCTAT	305	56	35

SYP	For: TGCAGAACAAGTACCGAGAG<br>Rev: CTGTCTCCTTAAACACGAACC	297	56	35
CACNA1C	For: ACCTGGAATGTCTGAAGCGA<br>Rev: TTTCTCACTGGACTCGACCC	248	60	30
IP3R1	For: GACCTTCGGGACGAAGAGAG<br>Rev: AATGCTTTCATGGAACACTCGGTC	230	60	30
IP3R2	For: AGCAACATCCAAAGCATATTGTGT<br>Rev: AATGCTTTCATGGAACACTCGGTC	198	59	30
RYR2	For: ATCCACAAAGAACAACAGAAGCTAT<br>Rev: CCATAAGACAAGTGCAAGTACCTTT	509	60	30
ACTN2	For: AGGAGGAAGAATGGCCTGAT<br>Rev: GATGCAGTACTGGGCCTGAT	291	60	30

Supplementary Figure 1

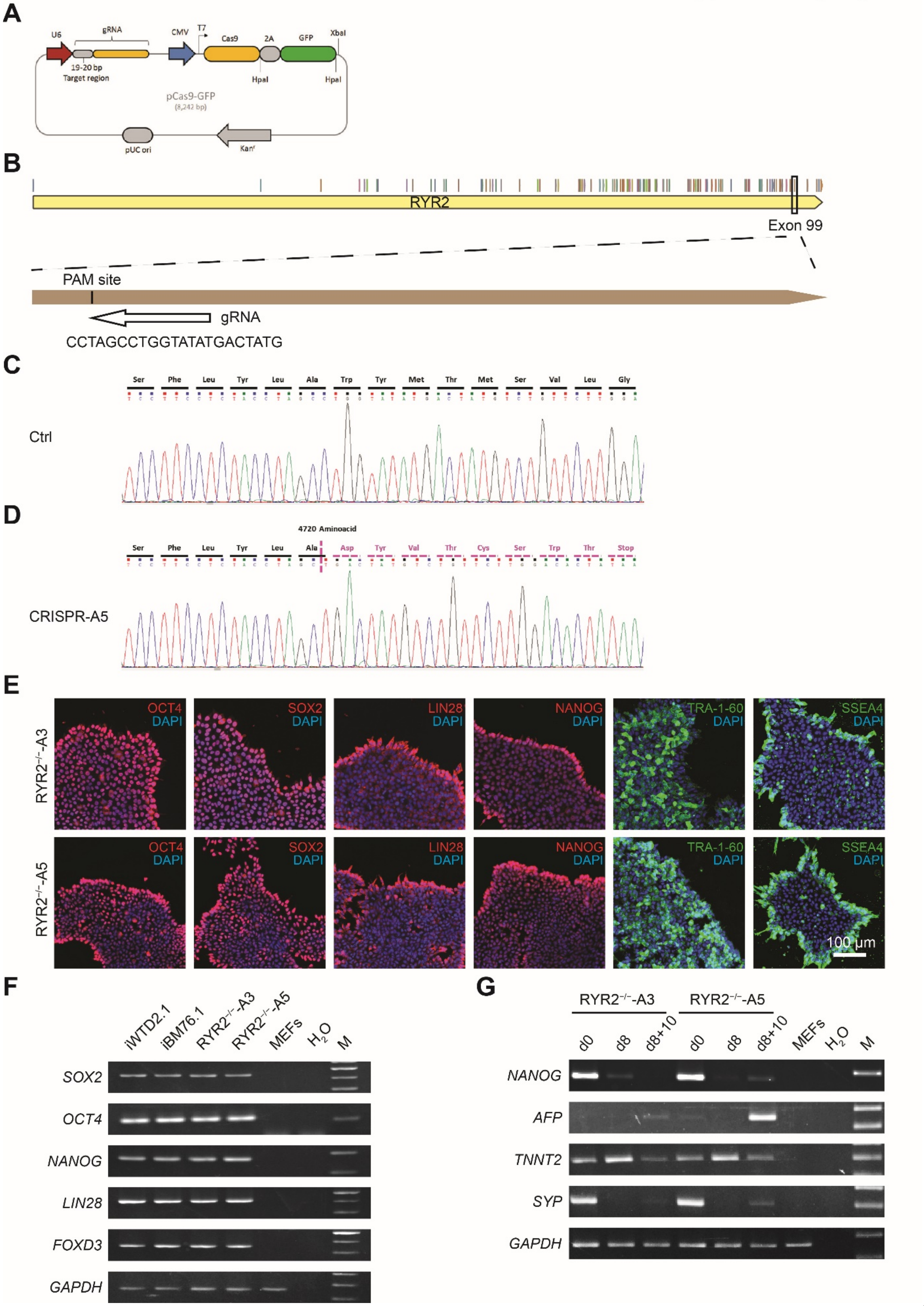

Supplementary Figure 1. DNA sequencing of CRISPR/cas9-edited A5 iPSCs and pluripotency characterization of A3 and A5 RYR2$^{-/-}$-iPSC lines. (A) CRISPR/Cas9 plasmid containing the sequence of gRNA, Cas9, and GFP. (B) Illustration of the *RYR2* locus and gRNA2 designed for CRISPR/Cas9-mediated gene editing in exon 99 of *RYR2*. (C, D) DNA sequencing of the Ctrl- and CRISPR/Cas9-edited A5 RYR2$^{-/-}$-iPSCs. CRISPR/Cas9-edited iPSC line A5 displayed a homozygous deletion of eight nucleotides in the *RYR2* gene, which resulted in a shift of the open reading frame and a premature termination codon. (E) Shown are immunofluorescence staining of the A3 and A5 RYR2$^{-/-}$-iPSC lines with respect to the pluripotency makers OCT4, SOX2, LIN28, NANOG, TRA-1-60, and SSEA4. Nuclei were stained with DAPI. Scale bar, 100 µm. (F) Reverse transcription-PCR analyses of RYR2$^{-/-}$-iPSC lines showing the endogenous expression of pluripotency genes (*OCT4, SOX2, LIN28, NANOG,* and *FOXD3*) similar to Ctrl-iPSCs. MEFs were used as a negative control, and *GAPDH* was used as a loading control. (G) Spontaneous differentiation capacity of the A3 and A5 RYR2$^{-/-}$-iPSC lines *in vitro*. The germ layer-specific genes *AFP, TNNT2,* and *SYP* were expressed in embryoid body outgrowths spontaneously differentiated from RYR2$^{-/-}$-iPSCs. Analyses were performed at differentiation stages (day 8, 8+10) during EB differentiation.

Supplementary Figure 2

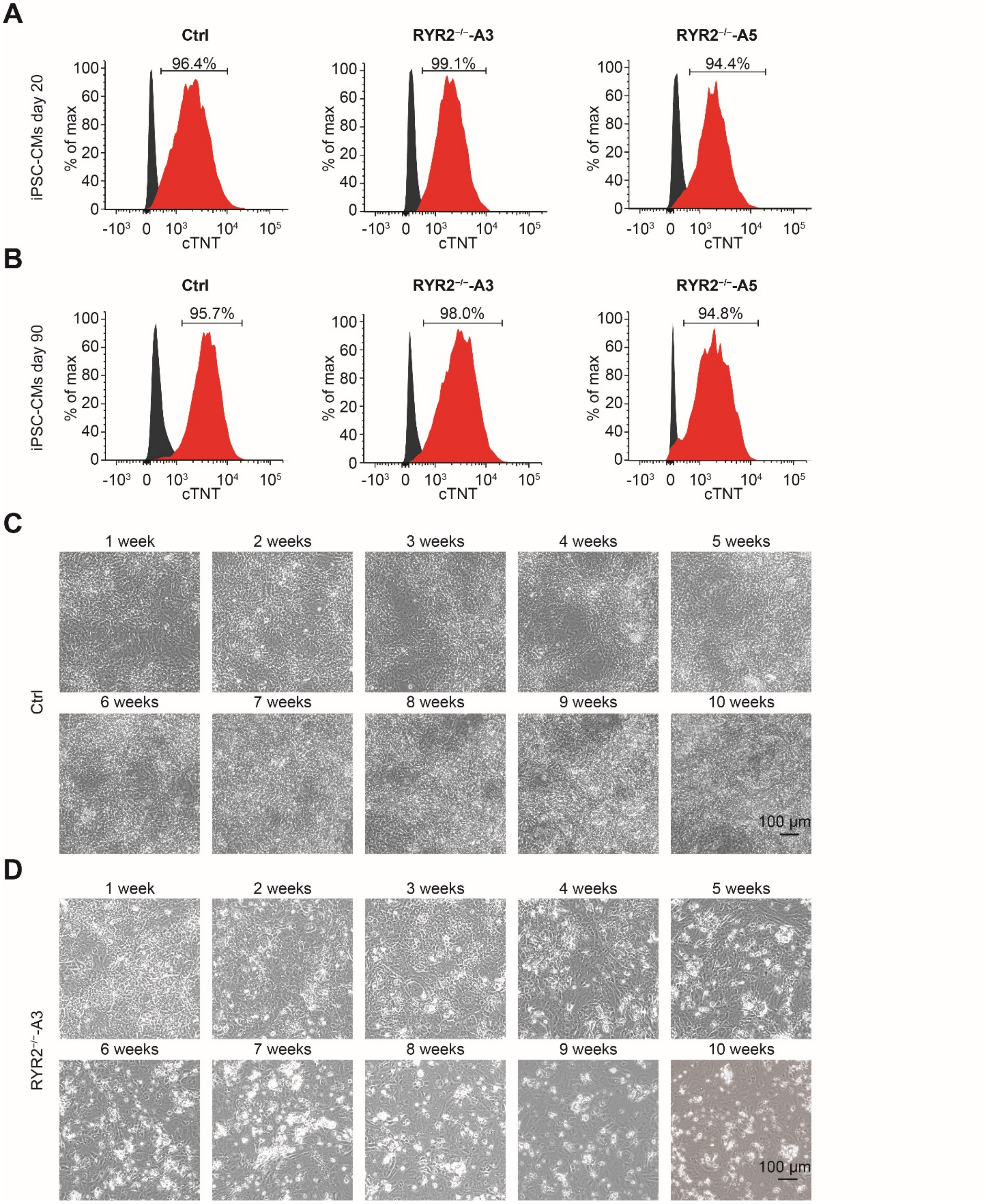

Supplementary Figure 2. Analysis of cTNT-positive iPSC-CMs and overview of iPSC-CM morphology during long-term culture. (A) Representative flow cytometry data show high population of cTNT-positive CMs in RYR2$^{-/-}$-iPSC-derived cells comparable to Ctrl-iPSC-derived

cells at day 20 post differentiation. (B) Purity of Ctrl- and RYR2$^{-/-}$-iPSC-CMs was similar after 10-week cultivation post replating. (C, D) Bright-field images of Ctrl- (C) and A3 RYR2$^{-/-}$-iPSC-CMs (D) from 1 to 10 weeks post replating. Scale bar, 100 μm.

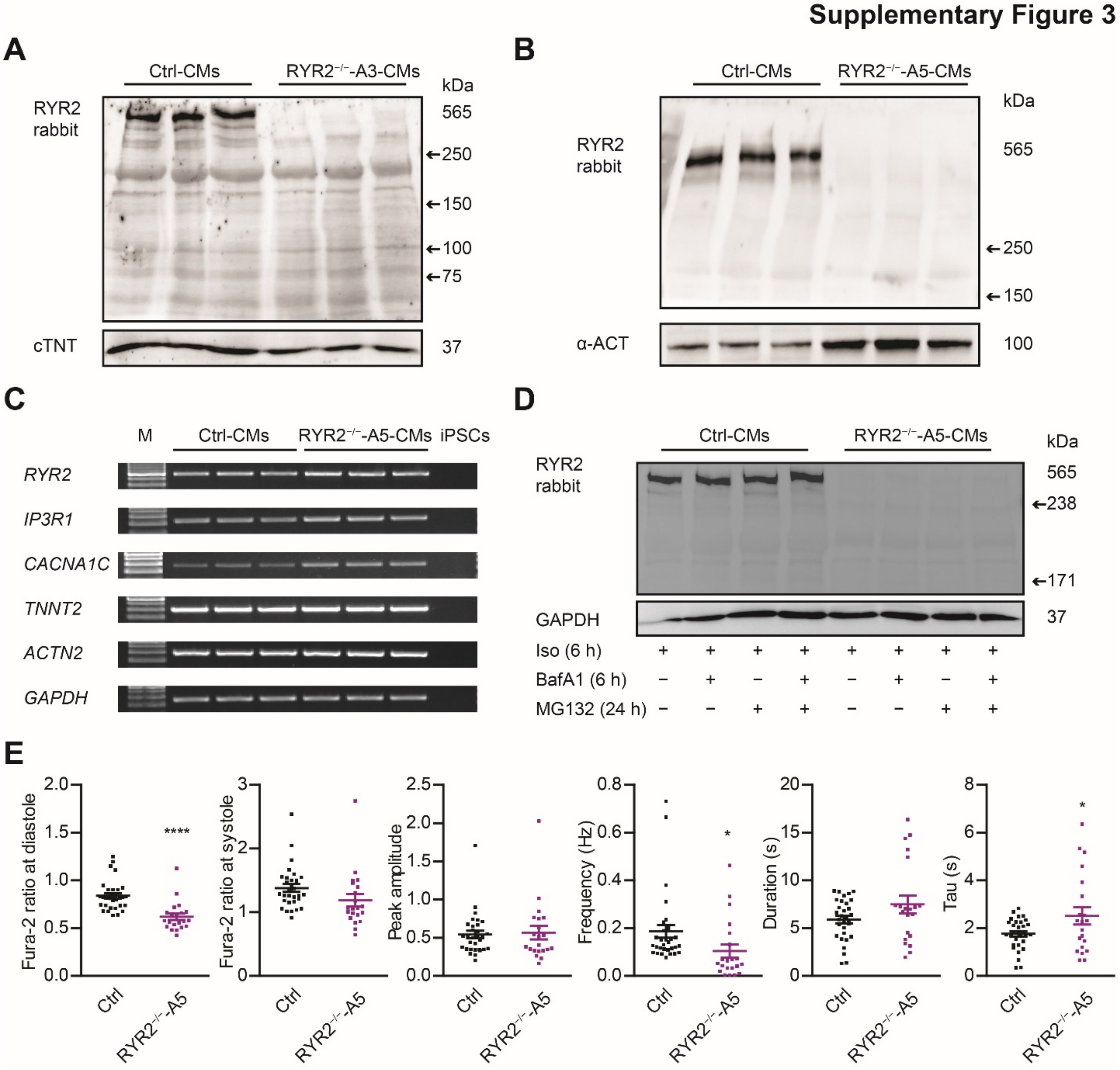

Supplementary Figure 3. RYR2 expression and Ca^{2+} transient analysis in Ctrl- and RYR2$^{-/-}$-iPSC-CMs. (A) Whole membrane of the western blot experiment shown in Fig. 3B. (B) Western blot analysis showing protein expression of N-terminal RYR2 and α-actinin (α-ACT) in Ctrl- and A5 RYR2$^{-/-}$-iPSC-CMs (Ctrl: n = 3 and A5 RYR2$^{-/-}$: n = 3 of independent differentiation experiments). (C) RT-PCR analyses showing mRNA expression of *RYR2, IP3R1, CACNA1C*, *TNNT2,* and *ACTN2*, and the housekeeping gene *GAPDH* in Ctrl- (n = 3) and A5 RYR2$^{-/-}$-iPSC-CMs (n = 3). (D) Western blot analysis of RYR2 in response to treatment with MG132 and/or Bafilomycin A (BafA1) in Ctrl- and A5 RYR2$^{-/-}$-iPSC-CMs (Ctrl: n = 2 and A5 RYR2$^{-/-}$: n = 2 differentiation experiment for each condition). No degraded RYR2 proteins were detected in A5

RYR2$^{-/-}$-iPSC-CMs by inhibiting protein degradation with the proteasome and calpain inhibitor MG132 and/or the autophagy inhibitor BafA1. (E) Characterization of spontaneous Ca^{2+} transients in Ctrl- and A5 RYR2$^{-/-}$-iPSC-CMs. Scatter dot plot shows the diastolic and systolic Ca^{2+} levels peak amplitude, frequency and duration of Ca^{2+} transients, as well as the time constant during the decay (tau) of Ca^{2+} transients between Ctrl- and A5 RYR2$^{-/-}$-iPSC-CMs (Ctrl: n = 31 cells from 5 differentiation experiments; A5 RYR2$^{-/-}$: n = 21 cells from 2 differentiation experiments). *P < 0.05; ****P < 0.0001 A5 RYR2$^{-/-}$ vs. Ctrl by Student's *t*-test.

Supplementary Video 1: Spontaneously beating A3 RYR2$^{-/-}$-iPSC-CMs at day 20 post differentiation.

Supplementary Video 2: Spontaneously beating of A5 RYR2$^{-/-}$-iPSC-CMs at day 20 post differentiation.

Supplementary Video 3: Spontaneously beating of Ctrl-iPSC-CMs at day 20 post differentiation.
